# Non-motor symptoms burden and motor recovery following STN–DBS in Parkinson's disease: a data-driven longitudinal analysis

**DOI:** 10.1007/s00415-026-14160-x

**Published:** 2026-10-01

**Authors:** Ghadir Abbas, Robert Peach, Uchralt Temuulen, Yunyou Tang, Tanmoy Sil, Anna Kufner, Matthias Endres, Jens Volkmann, Florian Lange, Martin Reich

**Affiliations:** 1 Klinik für Neurologie mit Experimenteller Neurologie, Charité – Universitätsmedizin Berlin, Corporate Member of Freie Universität Berlin and Humboldt-Universität zu Berlin, 10117 Berlin, Germany; 2 Center for Stroke Research Berlin (CSB), Charité – Universitätsmedizin Berlin, Corporate Member of Freie Universität Berlin and Humboldt-Universität zu Berlin, 12203 Berlin, Germany; 3 Berlin Institute of Health at Charité – Universitätsmedizin Berlin, 10117 Berlin, Germany; 4 German Center for Cardiovascular Research (Deutsches Zentrum für Herz Kreislauferkrankungen, DZHK), Partner Site Berlin, 10117 Berlin, Germany; 5 NeuroCure Clinical Research Center (NCRC), Charité – Universitätsmedizin Berlin, Corporate Member of Freie Universität Berlin and Humboldt-Universität zu Berlin, NeuroCure Cluster of Excellence, 10117 Berlin, Germany; 6 German Center for Neurodegenerative Diseases (Deutsches Zentrum für Neurodegenerative Erkrankungen, DZNE), Partner Site Berlin, 10117 Berlin, Germany; 7 Neurologische Klinik und Poliklinik, Universitätsklinikum Würzburg, Josef-Schneider-Straße 11, Haus B1, 97080 Würzburg, Germany

**Keywords:** Deep brain stimulation, Subthalamic nucleus, Parkinson's disease, Non-motor symptoms, Gait

## Abstract

**Background:**

Subthalamic nucleus–deep brain stimulation (STN–DBS) reliably improves cardinal motor symptoms in Parkinson's disease (PD). However, gait disorders and non-motor symptoms (NMS) may persist or emerge following surgery, diminishing quality of life and complicating clinical management.

**Objectives:**

To characterize the dominant axes of clinical covariance across motor and non-motor domains before and after STN–DBS, and to determine whether non-motor symptom severity moderates post-DBS gait and mobility change.

**Methods:**

100 PD patients undergoing STN–DBS (mean age 60.2 ± 7.6 years; 67% male) were studied. Principal component analysis (PCA) was performed on thirteen motor and non-motor sub-scores at pre-DBS, 1-year follow-up (1-y FU), and a subset (*n* = 33) at 3–5-year follow-up. Moderation was tested using analysis of covariance (ANCOVA): each 1-year motor outcome was regressed on its pre-DBS baseline, a non-motor moderator, and their interaction, adjusting for age and disease duration, with a significant interaction indicating that baseline non-motor severity was associated with the degree of subsequent motor recovery.

**Results:**

PCA identified a core motor triad: gait, mobility, and freezing of gait as the primary source of clinical variance at all timepoints (PC1: 35% pre-DBS, declining to 28% at 3–5-y FU), PC1 was the only component to exceed a permutation null (*p* < 0.001), with memory-related impairment increased at longer-term follow-up. Moderation analysis showed that higher pre-operative mood burden specifically for psychomotor and psychosomatic symptoms were associated with poorer recovery of mobility (Psychomotor *β* = 1.16, *p* < 0.05). At 1-y FU anxiety and psychosomatic symptoms showed concurrent associations with mobility and overall motor burden (Anxiety *β* = 2.30, *p* < 0.05).

**Conclusions:**

STN–DBS outcomes extend beyond global motor improvement and may be shaped by associations spanning the motor, cognitive, and mood domains. Higher NMS baseline burden was associated with poorer mobility recovery, and mood symptoms at follow-up were associated with concurrent motor burden. These exploratory findings support considering multi-domain pre-operative assessment and post-operative mood monitoring, as potential targets for optimizing motor outcomes.

**Supplementary Information:**

The online version contains supplementary material available at https://doi.org/10.1007/s00415-026-14160-x.

## Background

Parkinson’s disease (PD) is a progressive neurodegenerative disorder characterized by a range of motor symptoms, including bradykinesia, tremor, rigidity, and gait disturbances, as well as diverse non-motor symptoms (NMS) [1, 2], including mood disorders (40% prevalence) and anxiety disorders (31% prevalence) [3, 4], and cognitive decline, such as mild cognitive impairment (MCI; 20–50% prevalence in later disease stage) [5].

Subthalamic nucleus–deep brain stimulation (STN–DBS) is an evidence-based surgical intervention for PD, that effectively reduces cardinal motor symptoms of PD, particularly bradykinesia and tremor while also reducing the dependence of dopaminergic medication [6–8]. However, it has limited efficacy in restoring complex motor functions, such as gait [9–11]. In addition, STN–DBS can substantially modulate non-motor symptoms associated with PD, including cognitive aspects—memory, verbal fluency, and attention—as well as mood and anxiety [2, 9, 12–14].

The interplay between motor and non-motor symptoms in PD, and how this interplay is affected by STN–DBS, has attracted increasing research interest. NMS substantially impacts patients’ quality of life and has a large impact on key motor outcomes in PD, such as gait and freezing of gait (FoG) [2]. For example, anxiety is associated with higher rates of FoG [15], and executive dysfunction can negatively impact mobility and gait quality, increasing the risk of falls [16].

Suboptimal outcomes for certain motor and non-motor features following STN–DBS present challenges for clinicians and can lead to patient dissatisfaction [17]. Many patients report that persistent or worsening gait disorders and NMS significantly diminish their quality of life, despite overall motor improvement [18, 19]. This often means their needs related to daily life functions, including occupational and leisure activities, remain unfulfilled [20].

Although the interplay between motor and non-motor domains following STN–DBS has been examined previously, prior work has largely addressed selected, pairwise relationships rather than the joint structure across domains. Early analyses correlated summary scores of motor and cognitive/affective change but reported no significant associations [21]. Long-term follow-up studies tracked motor and cognitive outcomes over up to 8–15 years yet analyzed each domain separately rather than modeling their interdependence [22]. Dual-task paradigms have shown that STN–DBS does not improve gait under cognitive load, highlighting a motor–cognitive interaction that stimulation leaves unresolved [23]. More recently, cohort studies have linked domains directly: frontal-executive dysfunction and older age at disease onset predict axial decline and frequent falls over long-term stimulation [24], and mood and cognitive burden interact with motor severity to shape fall risk [25]. Collectively, these studies establish that cross-domain interactions after STN–DBS are clinically meaningful, but they have characterized isolated associations or single outcomes; the broader covariance structure across motor, cognitive, and mood domains, and whether STN–DBS preserves or reconfigures it, remains uncharacterised.

Accordingly, this study investigated the associations between clinical features across three symptom domains: motor (with focus on gait and mobility), cognitive (e.g., memory, attention), and mood (e.g., anxiety, psychosomatic), and how these features and their relations change following STN–DBS. Our specific objectives were: (1) to characterize baseline multidomain clinical profiles and their evolution after STN–DBS using motor, cognitive, and mood features; (2) to characterize the dominant patterns of clinical covariance at each timepoint and to assess whether this covariance structure is preserved or changes following DBS; and (3) to examine whether baseline cognitive and mood characteristics are associated with heterogeneity in subsequent gait and mobility outcomes.

## Methods

### Participants

Patients with PD who underwent STN–DBS at the University Hospital Würzburg between 2010 and 2020 were included. Inclusion required: (1) confirmed PD diagnosis per DGN guidelines; (2) complete pre-operative assessments across all three domains; and (3) at least one post-operative follow-up at ~12 months. Patients with dementia, major psychiatric disorder, incomplete neurobehavioral assessment, or inconsistent outcome measures were excluded. All patients provided written informed consent for the scientific use of their clinical data at the time of treatment. The study was approved by the Ethics Committee of the University of Würzburg (248/20-me) in accordance with the Declaration of Helsinki.

### Clinical assessment

Data were collected at baseline (pre-DBS), 1-year follow-up (1-y FU), and 3–5-year follow-up (3–5-y FU). LEDD was calculated at pre-DBS and 1-y FU [26]. Motor function was assessed using MDS–UPDRS Parts II and III in MED–OFF state (overnight withdrawal of dopaminergic medication) pre-DBS and MED–OFF/STIM ON post-DBS at 1-y FU and 3–5-y FU [27]. Cognitive function was assessed with MoCA [28] or the Mattis Dementia Rating Scale (MDRS) [29], used consistently within each patient across timepoints. Mood was assessed with the HDRS [30]. Paired *t* tests were used for MDS–UPDRS II and III; Wilcoxon signed-rank tests for scales with ceiling/floor effects. All *p* values were FDR-corrected (Benjamini–Hochberg) across five main simultaneous comparisons.

To assess whether loss to long-term follow-up was selective, baseline and post-operative characteristics were compared between patients with and without a 3–5-year follow-up visit using Welch's *t* test or the Mann–Whitney *U* test for continuous variables (chosen according to distribution) and the chi-square test for sex.

### Overview of analytical pipeline

Three analyses were conducted sequentially: (1) clustering analysis to identify baseline clinical profiles; (2) principal component analysis (PCA) to identify the dominant patterns of clinical variation; and (3) moderation analysis to test cross-domain interactions as predictors of DBS outcome.

1. *Clustering analysis* Clustering was performed on 22 clinical sub-domain scores spanning motor, mood, and cognitive domains. Missing values were filled in (imputed) using multiple imputation by chained equations (MICE; scikit-learn IterativeImputer, round-robin conditional regression with a BayesianRidge estimator, median initialization, ≤ 25 iterations, converged in 4; seed 147), fitted only on pre-DBS data, so that information from follow-up visits could not bias the result. The primary clustering used a single completed dataset; to verify robustness to imputation uncertainty, 20 multiply-imputed datasets (sample_posterior = True; seeds 10,147–10166) were each carried independently through the identical standardization–PCA–k-means pipeline (check Online Resources; Supplementary Methods for more details). Features were then z-score standardized with a scaler also fit on pre-DBS data. PCA (sklearn.decomposition.PCA, Python 3.11) was applied to the standardized pre-DBS data, retaining all 22 components (explaining 99.9% of cumulative variance). Optimal cluster number was determined by silhouette scores (*k* = 2–10). Cluster stability was assessed by non-parametric bootstrap resampling (*B* = 500), quantifying the reproducibility of each cluster as the mean clusterwise Jaccard similarity between the original partition and partitions obtained on resampled data [31]. Each post-DBS (follow-up) observation was projected into the fixed baseline space: the imputation model, the standardization parameters (pre-DBS means and standard deviations), the PCA loadings, and the k-means cluster centroids were all estimated on pre-DBS data and applied unchanged to the follow-up data, with no parameter re-estimated at any follow-up timepoint. Follow-up cluster assignment was then determined by nearest baseline centroid.
2. *Dominant patterns of clinical covariation* A separate PCA was performed on 13 sub-domain scores (Online Resources; Table S1), excluding tremor to avoid masking gait- and mobility-related variance [32]. Bradykinesia and rigidity asymmetry scores (BAS, RAS) were computed as the absolute left–right MDS–UPDRS III differences to capture the magnitude of motor asymmetry [33]. For per-feature longitudinal comparisons, the normality of the paired differences was assessed with the Shapiro–Wilk test and a Wilcoxon signed-rank test used, where they departed from normality (paired t test otherwise), with Benjamini–Hochberg FDR correction. Sub-domain scores were then min–max normalized for the PCA analyses. To quantify within-patient longitudinal change on fixed clinical dimensions, the PCA was fitted on pre-DBS data only; the 1-year and 3–5-year observations were projected onto the fixed baseline loadings. Change in the projected component scores relative to baseline was tested with linear mixed-effects models (random intercept per patient; timepoint as a fixed effect, baseline as reference), across 233 patient visits (*n* = 100, 100, and 33 at pre-DBS, 1-year, and 3–5-year follow-up). The independent per-timepoint PCAs were retained as a secondary analysis of covariance-structure change (Procrustes disparity restricted to the first five principal components, against a permutation null, n_perm = 5000). As a sensitivity analysis, the effect of medication change (ΔLEDD) was statistically removed (residualised) from each post-DBS score, and the PCA was repeated on the adjusted scores.
3. *Moderation analysis* To test whether non-motor symptom severity was associated with the magnitude of post-DBS motor change while avoiding the coupling inherent in change-score models [34], the 1-year follow-up score for each motor outcome was regressed on its pre-DBS baseline value, the non-motor moderator, and the baseline × moderator interaction, adjusting for age and disease duration (ANCOVA: analysis of covariance). A significant interaction term indicated that the moderator modified the degree of recovery. All continuous predictors were min–max normalized to [0, 1]; both constituents of each interaction were mean-centered before the interaction term was formed, and the product term was not re-standardized. Reported coefficients are on this common [0, 1] scale (range-normalized, not z-standardized). Baseline (pre-DBS) non-motor severity was the primary moderator; 1-year follow-up non-motor severity was examined separately as a concurrent cross-domain association, as it is measured at the same visit as the outcome. For each motor outcome at each timepoint, interaction p values were FDR-corrected (Benjamini–Hochberg) across the eight candidate non-motor moderators. Moderation models were fitted with HC3 heteroscedasticity-consistent robust standard errors.

### Artificial intelligence-generated content (AIGC)

The authors used Claude (Anthropic; Sonnet 4.6) to assist with code development for statistical analyses, and with editing of manuscript text. All outputs were reviewed and verified by the authors, who are fully responsible for the content and accuracy of the manuscript.

## Results

### Patient characteristics

Of 180 consecutive Würzburg patients who underwent STN–DBS between 2010 and 2020, 100 met all inclusion criteria (see Online Resources; Fig. S1). The final sample comprised 100 patients (mean age at surgery: 60.2 ± 7.6 years; range 42–87; 67 males, 33 females). A subset of 33 patients had complete assessments at 3-y or 5-y follow-up (Online Resources; Tables S2 and S3).

### General clinical assessment

**Fig. 1:**
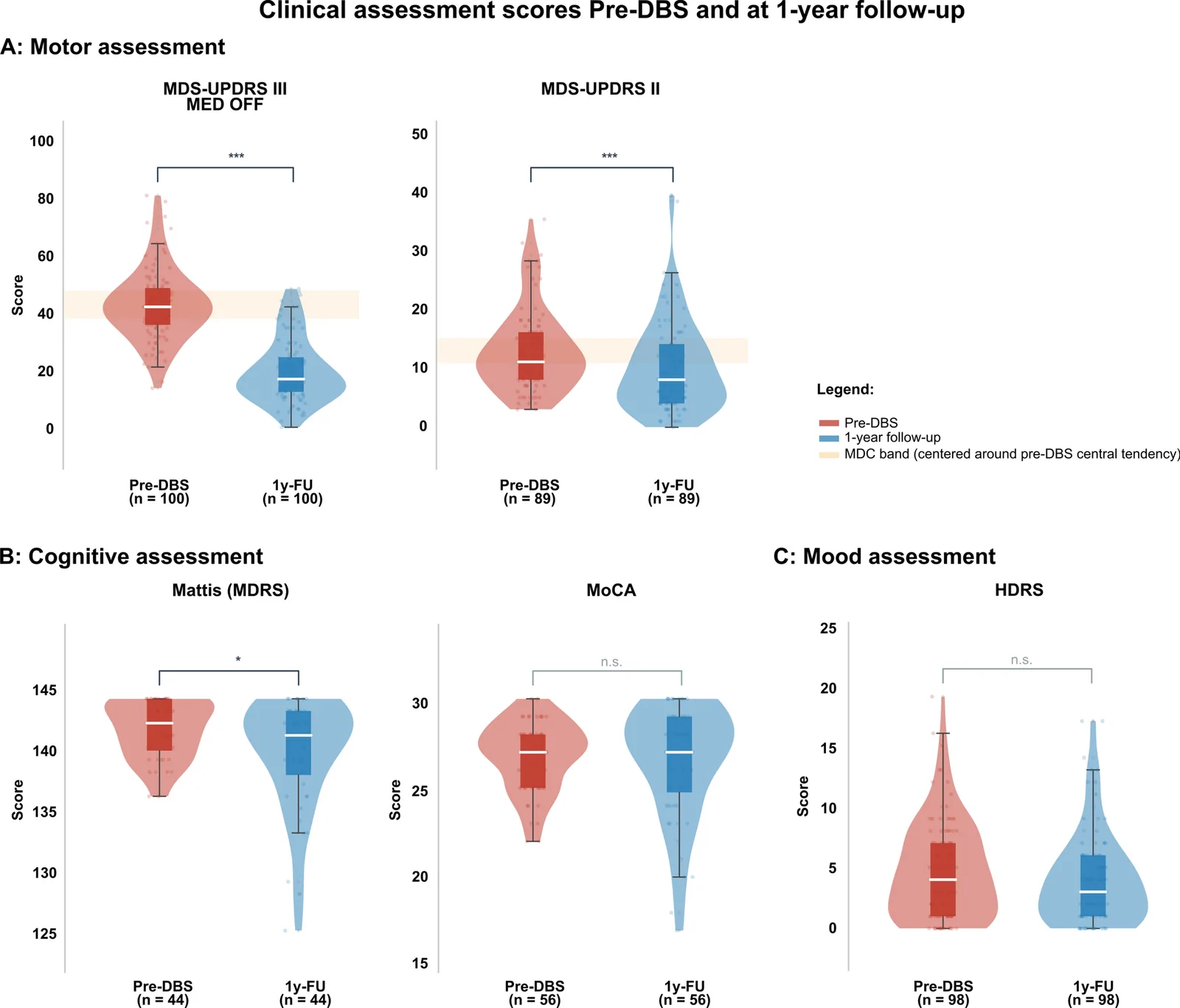
Clinical assessment scores before and at 1 year after STN–DBS (*n* = 100, MED OFF). Violin plots with superimposed box plots and jittered individual data points showing score distributions at pre-DBS baseline (red) and 1-year follow-up (blue). The orange band indicates the minimal detectable change (*MDC*) window centered on the pre-DBS central tendency. **A** STN–DBS induced significant improvements in motor function: MDS–UPDRS II (*t* = 3.96, *p* < 0.001), and MDS–UPDRS III (*t* = 18.3, *p* < 0.001) both exceeding the MDC threshold. **B**, **C** Non-motor outcomes did not change significantly: HDRS, and MoCA showed no significant change after FDR correction, while the Mattis score showed a statistically significant decline. Wilcoxon signed-rank tests were used as the primary test for scales with ceiling or floor effects (Mattis, MoCA, HDRS); paired *t* tests for MDS–UPDRS II and III. All *p* values are FDR-corrected (Benjamini–Hochberg, *k* = 5). *** *p* < 0.001; * *p* < 0.05; *n.s.* not significant

Table 1 summarizes statistical testing for each of the main assessment scales, also for relevant motor subscales (Mobility, gait, FoG, BAS, RAS) between pre-DBS and 1-y FU. Paired *t* tests with FDR correction revealed significant improvement in motor function as measured by MDS–UPDRS-II (*t* = 3.96, *p* < 0.001) and MDS–UPDRS-III (MED OFF) (*t* = 18.03, *p* < 0.001). A modest but statistically significant decline was observed on the Mattis scale (*W* = 160, *p* < 0.05), assessed in a smaller subset (*n* = 44). MoCA scores did not change significantly (*W* = 552.5, *p* > 0.05; *n* = 56), nor did mood scores on the HDRS (*W* = 1495.5, *p* > 0.05). Data distributions are shown in Fig. 1.

**Table 1:** Main clinical scale and motor sub-scale comparisons (pre-DBS vs 1-year follow-up)

	Pre-DBS		1-y FU		Statistic (FDR *p*)
Scale	*N*	Mean (± SD)	*N*	Mean (± SD)	
Sum MDS–UPDRS-II	94	12.89 ± 6.87	92	10.09 ± 7.63	*t* = 3.96 (*p* < 0.001)
Sum MDS–UPDRS-III (MED OFF)	100	43.68 ± 11.96	100	21.60 ± 10.50	*t* = 18.03 (*p* < 0.001)

Scale	*N*	Median [IQR]	*N*	Median [IQR]	
Sum Mattis	44	142.0 [139.8–144.0]	44	141.0 [137.8–143.0]	*W* = 160.0 (*p* < 0.05)
Sum MoCA	56	27.0 [25.0–28.0]	56	27.0 [24.8–29.0]	*W* = 552.5 (*p* > 0.05)
Sum HDRS	100	4.0 [1.0–7.0]	98	3.0 [1.0–6.0]	*W* = 1495.5 (*p* > 0.05)

Motor sub-scale (MED–OFF)	*N*	Median [IQR]	*N*	Median [IQR]	
Mobility	93	3.0 [2.0–4.0]	92	1.0 [0.0–3.0]	*W* = 405.5 (*p* < 0.001)
Gait	96	4.0 [2.0–6.0]	94	2.0 [1.0–4.8]	*W* = 442.0 (*p* < 0.001)
Freezing of gait	96	2.0 [0.0–3.2]	94	0.5 [0.0–2.0]	*W* = 335.0 (*p* < 0.001)
Bradykinesia asymmetry (BAS)	99	3.0 [1.0–4.0]	99	2.0 [1.0–3.0]	*W* = 1277.5 (n.s.)
Rigidity asymmetry (RAS)	100	1.0 [1.0–2.0]	99	1.0 [0.0–1.5]	*W* = 847.5 (n.s.)

For each measure, values are given at pre-DBS baseline and at 1-year follow-up, with the number of patients assessed (*N*) at each timepoint. Motor function (MDS–UPDRS Parts II and III) and all motor sub-scales were assessed in the MED–OFF state; MDS–UPDRS-III and the motor sub-scales at follow-up were additionally in the stimulation-ON condition. Normally distributed measures (MDS–UPDRS-II and -III) are summarized as mean ± SD and compared with paired *t* tests; measures with floor/ceiling effects or otherwise non-normal distributions (Mattis, MoCA, HDRS, and all motor sub-scales) are summarized as median [interquartile range] and compared with Wilcoxon signed-rank tests. *p* values for the summary scales were false-discovery-rate corrected (Benjamini–Hochberg) across the five scales; *p* values for the motor sub-scales are drawn from the per-feature longitudinal analysis (FDR-corrected across that analysis; Online Resources: Table S7 and Fig. S3), where the complete distributions of all sub-domain scores across timepoints are also reported.

*n.s.* not significant (*p* > 0.05), *BAS* bradykinesia asymmetry score, *FDR* false discovery rate, *HDRS* Hamilton Depression Rating Scale, *IQR* interquartile range, *MDS–UPDRS* Movement Disorder Society–Unified Parkinson's Disease Rating Scale, *Mattis* Mattis Dementia Rating Scale, *MoCA* Montreal Cognitive Assessment, *RAS* rigidity asymmetry score, *SD* deviation

Patients retained at long-term follow-up (*n* = 33) did not differ from those without a 3–5-year visit (*n* = 67) in age, sex, disease duration, MDS–UPDRS-II, post-operative MDS–UPDRS-III, or LEDD (all FDR-adjusted *p* > 0.05). Pre-operative MDS–UPDRS-III was higher in the retained group (median [IQR]: 46 [43.0 - 55.0] vs 40 [35.0 - 47.0] ; adjusted *p* = 0.029; Online Resources; Table S4).

### Clustering PD patients using sub-domains as features

Unsupervised k-means clustering of pre-DBS clinical profiles identified two optimal clusters based on the silhouette score (*k* = 2: score = 0.124; all *k* > 2: score < 0.124) described hereafter as higher- and lower-impairment clinical profiles. A non-parametric bootstrap stability analysis (*B* = 500) supported the two-cluster solution, which was the only partition to avoid dissolution (mean clusterwise Jaccard = 0.77; all *k* ≥ 3: 0.38–0.49). Both clusters were moderately stable under Hennig's criterion (mean Jaccard 0.81 and 0.73), indicating reproducible but not highly distinct subgroups (Online Resources; Table S5).

Cluster assignments were robust to imputation: across 20 multiply-imputed datasets the mean Adjusted Rand Index with the primary solution was 0.945 (SD 0.047; minimum 0.845) and mean per-patient assignment stability was 0.988, with only 2/100 patients sensitive to imputation choice.

**Fig. 2:**
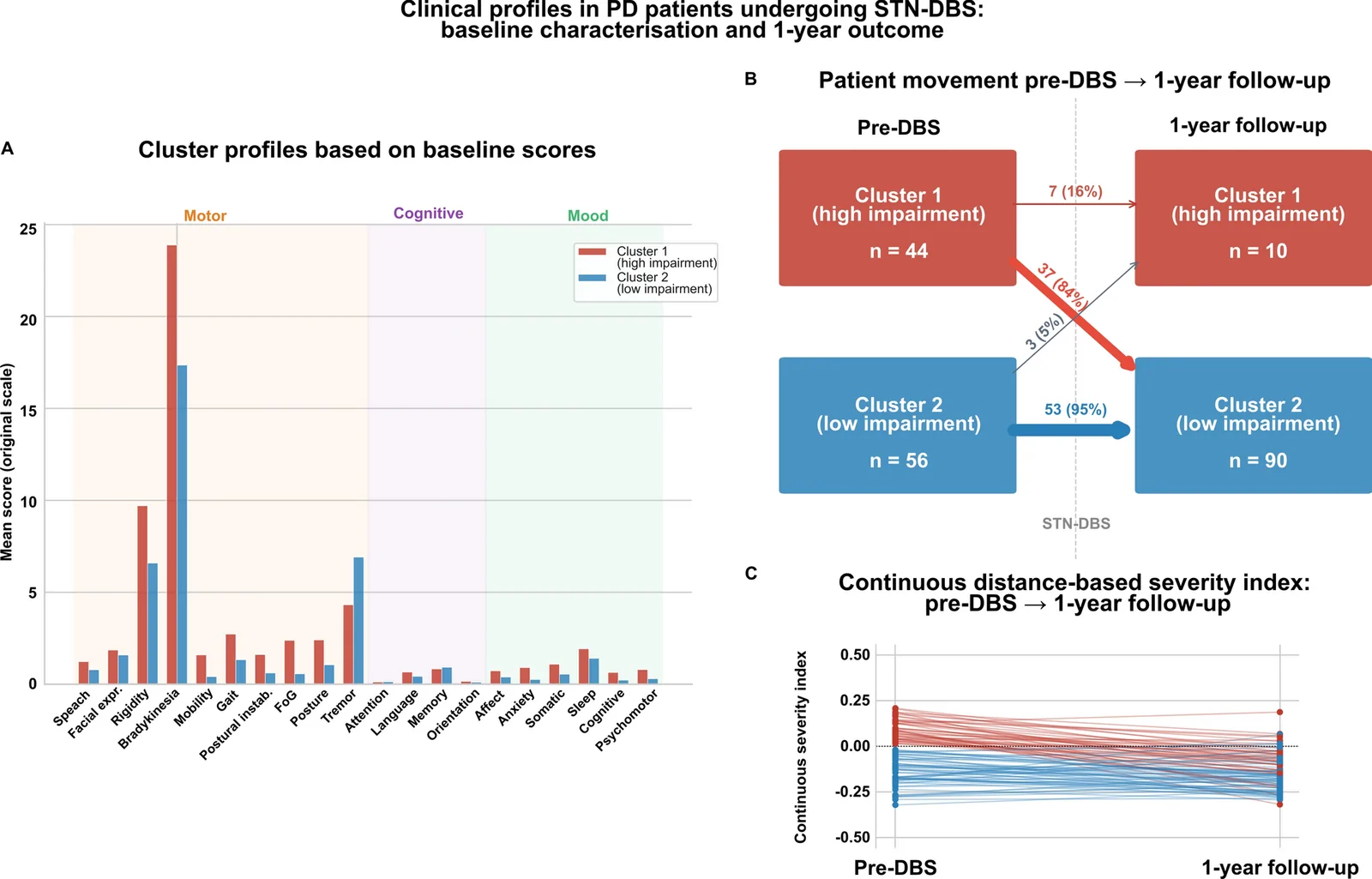
Clinical profiles in PD patients undergoing STN–DBS: baseline characterization and 1-year outcome. **A** Mean feature scores at pre-DBS baseline for Cluster 1 (high impairment, *n* = 44, red) and Cluster 2 (low impairment, *n* = 56, blue) across motor, cognitive, and mood domains. The two profiles were differentiated primarily by motor severity, particularly bradykinesia, rigidity, and gait-related features, with minimal separation in the cognitive domain. **B** Changes in profile assignment from pre-DBS to 1-year follow-up, with post-DBS assignments determined by nearest-centroid projection into the pre-DBS cluster space. Of 44 high-impairment patients, 37 (84%) transitioned to the low-impairment profile, and 7 (16%) remained; of the 56 lower-impairment patients, 53 (95%) remained and 3 (5%) moved to the higher-impairment profile. Percentages are row-normalized (i.e., relative to each baseline profile), and arrow thickness is proportional to patient count. **C** Continuous distance-based severity index for each patient at pre-DBS and 1-year follow-up. The index locates each patient along the baseline severity axis, derived from the relative Euclidean distance to the two baseline profile centroids, with higher values indicating proximity to the higher-impairment centroid; lines connect repeated measures within a patient (red = higher-impairment profile, blue = lower-impairment profile). Most patients show a reduction in the index after DBS, most pronounced in the higher-impairment profile, indicating that the observed movement reflects improvement along a continuous severity dimension rather than discrete transition between subtypes

The two clusters differed most on the axial and appendicular motor sub-domains that dominate PC1 (Online Resources; Fig. S2): the largest separations were in postural instability (Cluster 1 vs Cluster 2: 2.39 ± 1.06 vs 0.57 ± 0.83; Cohen's *d* = 1.94), gait (2.73 ± 0.92 vs 1.33 ± 0.72; *d* = 1.72), posture (2.41 ± 0.82 vs 1.06 ± 0.82; *d* = 1.66), mobility (1.59 ± 1.30 vs 0.41 ± 0.53; *d* = 1.24), bradykinesia (23.89 ± 6.22 vs 17.36 ± 5.59; *d* = 1.11), and rigidity (9.70 ± 3.37 vs 6.59 ± 3.17; *d* = 0.96). Differences in the mood sub-domains were smaller (anxiety *d* = 0.78; psychomotor and psychosomatic *d* = 0.54–0.58; affect *d* = 0.35), whereas tremor was slightly higher in the lower-impairment cluster (4.32 ± 5.06 vs 6.91 ± 6.23; *d* = −0.45). These differences are descriptive and expected by construction, since the same variables (via PCA) generated the partition (Online Resources; Table S6). Mean scores for each feature in the original scales are shown in Fig. 2A. At 1-year follow-up, post-DBS clinical profiles were projected into the pre-DBS cluster space using the pre-DBS centroids. Of the 44 patients initially classified in the high-impairment profile, 37 (84%) moved to the low-impairment profile, while 7 (16%) remained in the high-impairment profile. Of the 56 patients initially in the low-impairment profile, 53 (95%) remained stable and 3 (5%) moved to the high-impairment profile (Fig. 2B). Because the profiles are separated primarily along a continuous severity dimension (PC1), this post-operative movement is most parsimoniously interpreted as improvement along that dimension rather than transition between distinct phenotypes, we, therefore, report the change in each patient's distance to the baseline centroids as a continuous descriptor of this movement (Fig. 2C).

### Gait and mobility dominate between-patient clinical variance across time

**Fig. 3:**
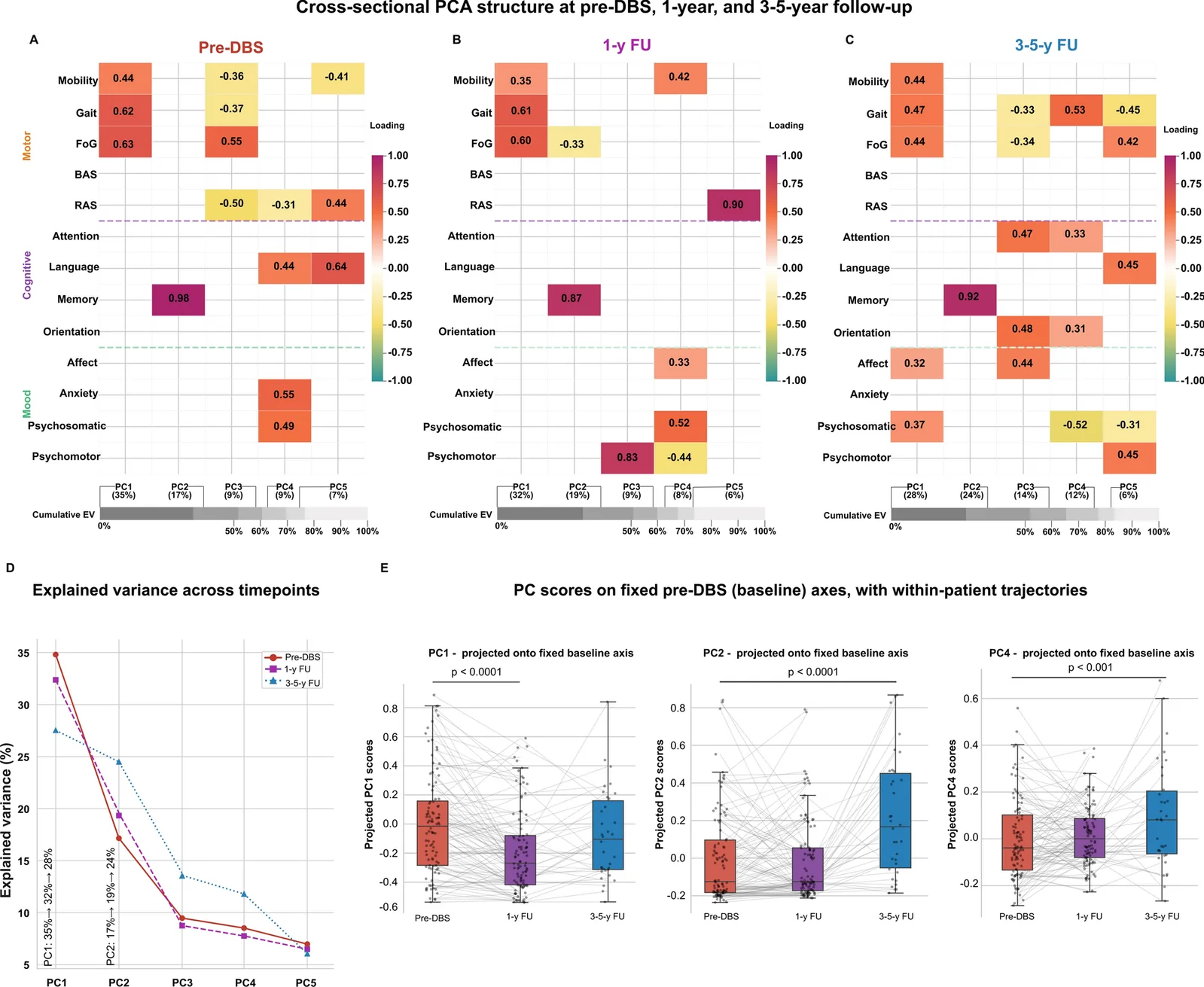
PCA structure at pre-DBS, 1-year, and 3–5-year follow-up. **A**–**C** Cross-sectional PCA loading heatmaps for the first five principal components at pre-DBS (**A**), 1-year follow-up (**B**), and 3–5-year follow-up (**C**), each computed on the 13 features min–max scaled to [0, 1]. Only loadings with |loading|≥ 0.25 are shown; features are grouped by domain (motor, cognitive, mood; dashed lines), and cumulative explained variance per component is shown beneath each heatmap. At all timepoints, PC1 was dominated by gait, FoG, and mobility (loadings 0.44–0.63) and PC2 by memory (loadings 0.87–0.98); higher-order components showed more variable, less stable contributions from mood and asymmetry features. **D** Explained variance per component across timepoints. The between-patient variance captured by PC1 decreased over time (35% pre-DBS → 32% at 1-y FU → 28% at 3–5-y FU) and that captured by PC2 increased (17% → 19% → 24%), reflecting a change in the relative between-patient heterogeneity of the motor and memory domains rather than a change in absolute clinical severity (quantified in E). **E** Within-patient longitudinal change on fixed baseline axes. Box plots show the resulting projected PC scores at each timepoint, with gray lines connecting repeated observations within patients. Change relative to baseline was tested with linear mixed-effects models (random intercept per patient; 234 patient visits). Projected PC1 scores (the baseline motor–severity axis) decreased significantly from baseline to 1-year follow-up (*p* < 0.0001), consistent with a reduction in motor severity; projected PC2 scores (memory) increased significantly at 3–5 years (*p* < 0.0001); projected PC3 scores increased modestly at 1 year (*p* < 0.001). BAS = bradykinesia asymmetry score; *FoG* freezing of gait, *PC* principal component, *RAS* rigidity asymmetry score

Distributions of the individual sub-domain scores (mean, SD, skewness) are reported in Online Resources; Table S7. Cross-sectional PCA on the targeted 13-feature set, fitted independently at each timepoint, characterized the between-patient covariance structure at each visit. Three components explained ≥ 60% cumulative variance at all timepoints (pre-DBS: 61.5%; 1-y FU: 60.5%; 3–5-y FU: 65.5%; Fig. 3A–C). Across all timepoints, PC1 was consistently dominated by gait, FoG, and mobility (‘PC-motor’), explaining 35%, 32%, and 28% of variance at pre-DBS, 1-y FU, and 3–5-y FU, respectively. PC2 was dominated by memory (loading ≥ 0.87); the proportion of between-patient variance it captured increased from 17 to 24%, reflecting a change in the relative between-patient heterogeneity of each domain rather than as changes in absolute clinical severity, which we quantify separately below (Fig. 3D). In a permutation (parallel) analysis, only PC1 exceeded the chance null (*p* = 0.001; PC2–PC5, *p* ≥ 0.60), indicating that PC2 and higher components represent largely domain-specific, near-independent variation rather than robust multivariate axes. Procrustes analysis restricted to the first five components indicated that the loading structure was significantly more aligned across timepoints than expected under a permutation null (5000 permutations): pre-DBS vs 1-y FU disparity = 0.47 (null 0.70, *p* = 0.006); pre-DBS vs 3–5-y FU = 0.47 (*p* = 0.006); 1-y FU vs 3–5-y FU = 0.50 (*p* = 0.009), indicating preservation of the dominant covariance structure across time rather than trivial alignment.

To quantify within-patient change on fixed clinical dimensions, the PCA was fitted on pre-DBS data only and the 1-year and 3–5-year observations were projected into this fixed baseline space; change in the projected component scores was tested with linear mixed-effects models (random intercept per patient; 233 patient-visits). Projected PC1 scores (the baseline motor–severity axis) decreased significantly from baseline to 1-year follow-up (β = −0.19, 95% CI [− 0.25, −0.13], *p* < 0.001) and returned toward baseline by 3–5 years (*β* = −0.08, *p* = 0.085), consistent with an initial reduction in motor severity followed by partial attenuation. Projected PC2 scores (memory) were unchanged at 1 year (*β* = −0.01, *p* = 0.67) but increased significantly at 3–5 years (*β* = +0.27, *p* < 0.001), consistent with an emerging memory-related burden over the longer term; PC4 (mood features) scores increased modestly at 3–5 years (*β* = +0.1, *p* < 0.001) (Fig. 3E). Per-feature analyses were consistent with the component-level findings: the gait, mobility, and freezing-of-gait sub-scores improved at 1-year follow-up, and language and memory declined at 3–5-year follow-up (Online Resources; Fig. S3).

In an exploratory subgroup analysis of patients with pre-existing MCI (*n* = 16; pre-DBS MoCA < 26 or Mattis < 136) the projected PC scores were subset to this subgroup and the identical linear mixed-effects models were re-fitted; because scores were projected onto the full-cohort PCA axes rather than re-estimated, the subgroup coefficients are on the same scale as, and directly comparable to, the full-cohort estimates. Only the pre-DBS-to-1-year contrast was estimable, as no patient in this subgroup had a long-term (3–5-year) follow-up visit. The 1-year reduction in PC1 (motor) scores seen in the full cohort was preserved in the MCI subgroup (*β* = −0.150, 95% CI [− 0.266, −0.034], *p* < 0.05; full cohort *β* = −0.188), consistent with comparable motor improvement. PC2 showed a decrease that was larger than in the full cohort (*β* = −0.165, 95% CI [− 0.354, 0.023], *p* = 0.085; full cohort *β* = −0.013) but did not reach significance. Given the subgroup size (*n* = 16), these estimates are underpowered and should be interpreted as exploratory (Online Resources; Fig. S4).

STN–DBS induced a significant reduction in LEDD (pre-DBS: median 1038 [IQR 725–1402] vs post-DBS: 475 [285–670] mg/day; Wilcoxon: *W* = 114, *p* < 0.001), corresponding to a median reduction of 525 mg/day (56.7%). To evaluate whether this medication change confounded the post-operative PCA structure, the effect of medication change (ΔLEDD) was statistically removed from each post-DBS score before repeating the PCA. The Procrustes distance between the original and LEDD-residualised 1-year follow-up PCA solutions was 0.004 (Online Resources; Fig. S5), indicating that the component structure was not materially altered by adjustment for medication change. Furthermore, no clinical change score was significantly correlated with ΔLEDD after FDR correction. However, because ΔLEDD is a post-treatment variable that may itself reflect the DBS response, this analysis cannot establish that the structure is DBS-related rather than medication-related.

### Cross-domain associations between non-motor burden and motor outcomes

**Fig. 4:**
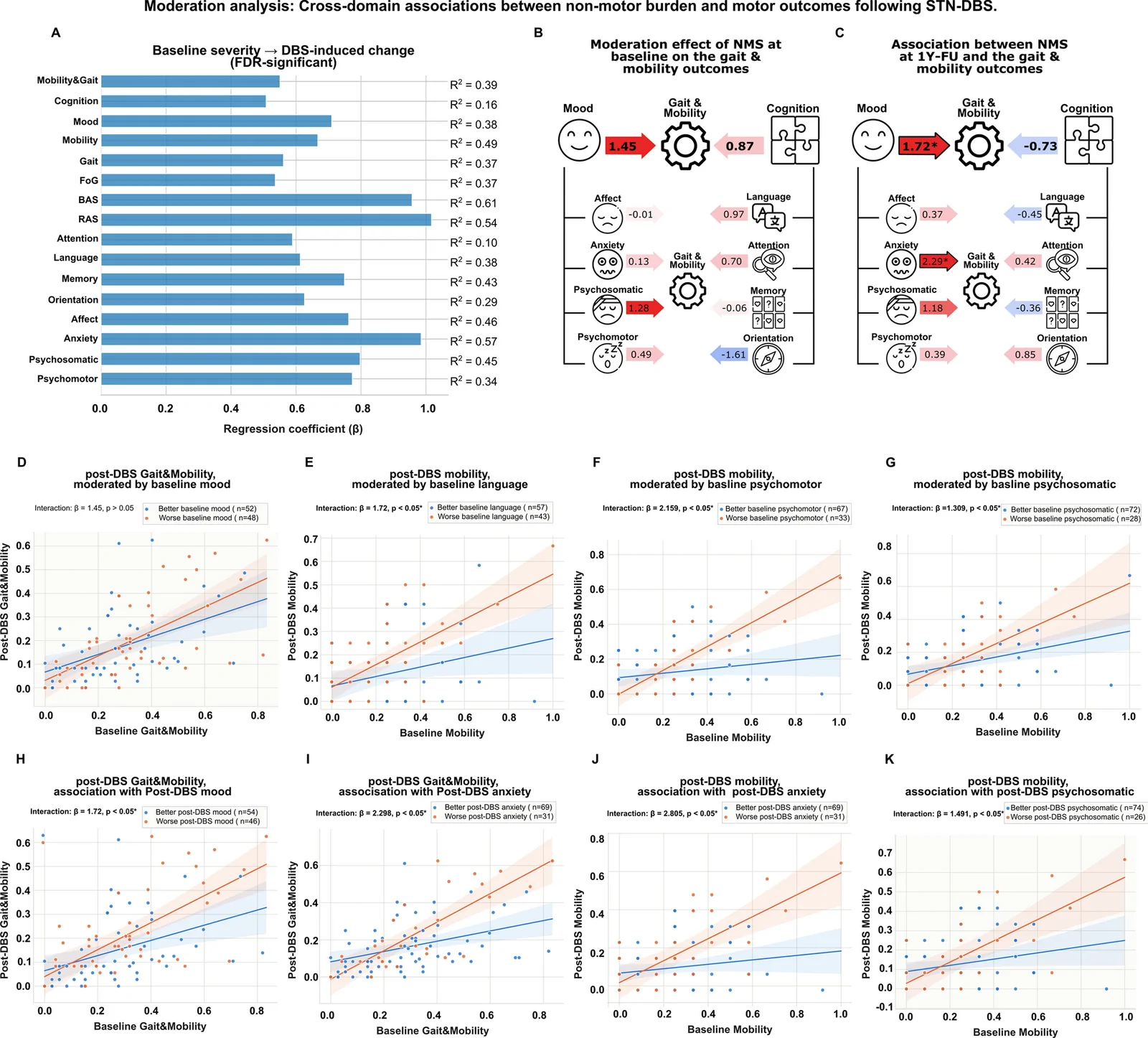
Cross-domain associations between non-motor burden and motor outcomes following STN–DBS. **A** Regression coefficients (min–max-normalized predictors) (*β*) from simple linear regressions of baseline severity on post-DBS change per feature (adjusted for age and disease duration; all FDR-significant; *R*^2^ annotated), reported descriptively. **B**, **C** Network diagrams of the baseline-adjusted analyses (ANCOVA): **B** baseline non-motor severity moderating gait/mobility recovery; **C** 1-year non-motor status as a concurrent, same-visit association (not a temporal moderator). Upper row, composite Mood/Cognition domains; lower rows, individual features. Arrow color = sign of the interaction coefficient (red = positive, i.e., poorer recovery with higher moderator severity; blue = negative). *FDR-corrected *p* < 0.05. **D**–**K** Simple-slope plots of the baseline → 1-year relationship, split by better (blue) vs worse (orange) moderator status (group n shown; 95% bands): baseline moderators—**D** mood [n.s.], **E** language, **F** psychomotor, **G** psychosomatic; concurrent 1 year—**H** mood, **I** anxiety, **J** anxiety, **K** psychosomatic. All models adjusted for age and disease duration; the moderation analyses are exploratory. *BAS/RAS* bradykinesia/rigidity asymmetry score, *FoG* freezing of gait, *NMS* non-motor symptoms

Simple linear regression showed that baseline severity was significantly associated with the subsequent post-DBS change for all motor and non-motor features (all *p* < 0.001 after FDR correction, Fig. 4A). Features with worse baseline scores showed greater improvement at 1-y FU. For example, mobility (*t* = 9.7, *R*^2^ = 0.49, *p* < 0.001), gait (*t* = 7.6, *R*^2^ = 0.37, *p* < 0.001), FoG (*t* = 7.5, *R*^2^ = 0.36, *p* < 0.001), BAS (*t* = 12.3, *R*^2^ = 0.60, *p* < 0.001) and RAS (*t* = 10.77, *R*^2^ = 0.54, *p* < 0.001) with a observations in the cognitive and mood domains. Complete results are provided in Online Resources; Table S8.

To test whether non-motor symptom burden moderated the post-DBS scores of the core motor triad identified by PCA—comprising gait, mobility, and FoG—we computed a composite motor triad score (sum of normalized gait, mobility, and FoG sub-scores) as outcome of interest (named Gait and Mobility in Fig. 4) and a composite NMS score (sum of normalized mood and cognitive sub-scores) as moderators. In baseline-adjusted models, higher baseline non-motor burden predicted poorer recovery of the composite motor triad (*β* = 1.45; Fig. 4B, D); to identify which specific NMS features drove these effects, we decomposed moderation into individual feature models. At baseline, psychosomatic depressive symptoms showed the strongest negative moderation effect on the Gait and Mobility outcomes (*β* = 1.28). We then looked in more details into the three motor triad components. Decomposing this into individual moderators, worse baseline psychomotor depressive symptoms (*β* = 2.16; Fig. 4F), and psychosomatic depressive symptoms (*β* = 1.31; Fig. 4G), and language function (*β* = 1.72; Fig. 4E), were associated with smaller mobility improvement, whereas better orientation was associated with greater improvement (*β* = − 2.17). We also examined the burden of NMS at 1-y FU as a concurrent cross-domain association rather than a temporal moderator (because it is measured at the same visit as the outcome); 1-year anxiety was associated with the composite motor score (*β* = 2.30; Fig. 4I) and with mobility (*β* = 2.81; Fig. 4J), as were psychosomatic depressive symptoms (*β* = 1.49; Fig. 4K). Gait and freezing of gait showed no FDR-significant moderators. These exploratory results highlight a possible negative role of the NMS specially mood and anxiety in the motor outcomes following the STN–DBS surgery, a finding that is yet to be confirmed in independent validation. A comprehensive list of all moderation outcomes is provided in Online Resources; Table S9. In addition, regression diagnostics [The RESET test (linearity), Cook's distance (influential observations), the Breusch–Pagan test (heteroscedasticity), and variance inflation factors (multicollinearity)] were computed for all models and are reported in Online Resources; Table S10.

## Discussion

This study used a data-driven, multi-method approach to characterize dominant axes of clinical variance before and after STN–DBS in PD. Our main findings were: (1) a severity axis derived from axial motor symptoms and mood features can separate patients into two clinical profiles, (2) a core motor triad of gait, mobility, and FoG consistently dominated between-patient clinical variance across all timepoints and, (3) pre-operative mood burden, particularly psychosomatic and psychomotor, significantly moderated mobility outcome post-DBS.

### Beyond the global motor improvement following STN–DBS

Unsupervised clustering identified two baseline clinical profiles differentiated primarily by motor and mood severity. The finding that 84% of high-impairment patients moved along the PC1 severity axis to the low-impairment profile at 1 year is consistent with the well-documented efficacy of STN–DBS in reducing motor burden [6, 8, 35], and with prior reports that DBS disproportionately benefits higher-impairment patients [8, 36] consistent here by higher baseline severity being associated with greater post-DBS change across all domains (Fig. 4A), and suggesting that baseline state of the patient could be used to determine the level of expected recovery.

Despite overall motor improvement, the persistent dominance of gait, mobility, and FoG across all PCA timepoints aligns with evidence documenting the limited effect of STN–DBS on axial motor features [9, 10, 37, 38], which remain among the most challenging unmet expectations of patients before surgery [17, 19, 20]. In addition, our results showed post-DBS improvement in motor function in the short term. We observed, however, that the derived PC1 scores, primarily reflecting motor function, increased at 3–5-year post-DBS, suggesting an attenuation of the initial motor improvements seen at 1-year follow-up (Fig. 3E), consistent with reports of > 40% initial gait improvement followed by significant long-term decline [9, 39, 40]. While this trajectory reflects the natural progression of dopaminergic and cholinergic degeneration [41–43], evidence suggests that adjusting stimulation settings or contacts can still partially address gait and FoG [44, 45].

### Non-motor symptoms after STN–DBS

STN–DBS has a complex impact on cognitive outcomes which might be attributed to the disease progression itself, dose of levodopa, or stimulation-related factors, including the precise electrode location and the settings of stimulation [46–49]. Memory-related scores increased over the longer term (within-patient projected PC2; Fig. 3E); because the memory component represented largely independent variation rather than a robust multivariate axis (permutation analysis), we interpret this as a domain-specific change in memory rather than a shift in overall clinical burden. This is consistent with the heterogeneous cognitive literature, where systematic reviews report either stability or modest global improvement, with the notable exception of verbal fluency [50–52]. Pre-existing cognitive conditions have been reported to play a determining role in the cognitive outcomes. Subgroup analysis of patients with MCI (n = 16) showed a non-significant trend toward memory improvement, similar to Ni et al. [53], but this is hypothesis-generating only given the sample size.

Mood features (PC4) showed no significant change at 1-y FU, but a small significant worsening at 3–5-y FU, consistent with reports of short-term improvement followed by later neuropsychiatric deterioration [54–56]. Despite this backdrop of modest and inconsistent cognitive and mood changes, our moderation analysis reveals a more specific and clinically relevant story: it is not whether mood improved, but whether pre-operative mood burden was high, that is associated with motor recovery.

### The interaction between motor and non-motor symptoms

Non-motor symptoms including memory deficits, attention, and anhedonia typically co-occur with motor symptoms from early disease stages [57, 58], and prior research has linked gait disorder and postural instability to anxiety, depression, and cognitive decline [15, 59, 60]. In our cohort, higher baseline non-motor burden was associated with poorer recovery of the motor triad (*β* = 1.45), the strongest individual associations for baseline psychomotor and psychosomatic depressive symptoms and language function was seen with mobility. Anxiety and psychosomatic depressive symptoms measured at 1 year were associated with mobility as a concurrent cross-domain relationship. The finding that higher pre-DBS psychomotor and psychosomatic depressive symptoms predicted higher mobility impairment post-DBS, together with the observation that anxiety and psychosomatic depressive symptoms at 1-y FU were associated with higher mobility burden, strengthens the case that pre-operative mood assessment is not merely prognostic for non-motor outcomes but could be directly relevant to expected motor benefit, and it hints post-operative mood management may be a modifiable target for optimizing motor recovery [61–63], a hypothesis that needs to be tested in a prospective cohort.

In addition, worse pre-DBS language function predicted higher mobility scores at 1-y FU (*β* = 1.72, *p* < 0.05), suggesting cognitive–motor interactions extending beyond the executive-gait pathways previously described [16], possibly reflecting frontal network dysfunction that limits the translation of DBS-induced circuit modulation into functional motor gains [13, 48]. We emphasize, however, that these analyses are exploratory, observational associations that provide the testable hypothesis that pre-operative mood and cognitive status are relevant to expected gait and mobility benefit.

### Possible mechanisms and suspected pathophysiology

One possible mechanism that could explain our results is the shared neural pathways underlying the motor and non-motor domains. The STN is involved not only in motor control but also in cognitive and limbic processes via striato-thalamo-cortical circuits [13]. The modulatory effects of DBS on cognitive and mood symptoms may, therefore, indirectly influence motor outcomes, particularly in domains, such as gait, mobility, and postural instability, which rely on cognitive-motor integration. In addition, it has been hypothesized that patients sacrifice cognitive performance to optimize gait and mobility [64], possibly because DBS-induced modulation of frontal circuits affects both cognitive control and postural adjustments [65], leading to unexpected mobility impairments despite overall motor improvements.

At the circuit level, symptom-specific tractography in a large multi-center DBS cohort demonstrated that axial motor symptoms—including gait and postural instability—are mediated by distinct white matter tracts connecting the STN to the supplementary motor cortex and brainstem, rather than the primary motor circuits most responsive to standard stimulation parameters [66]. This anatomical segregation may partly explain why gait and mobility remain the dominant axes of clinical variance even years after DBS, and suggests that the mood–motor interactions observed here may reflect shared involvement of prefrontal-subthalamic circuitry in both motor control and affective regulation [67].

Although dopaminergic medications [2, 68] are reported to have heterogeneous effects on different non-motor symptoms, the present analyses were conducted on MED OFF assessments, which minimizes confounding from acute dopaminergic medication effects. However, STN–DBS typically enables substantial reductions in levodopa equivalent daily dose, and chronic changes in dopaminergic tone may influence non-motor outcomes indirectly. As a sensitivity analysis to the robustness of our results for medication change, we showed that ΔLEDD did not correlate with the level of post-DBS change on all the motor and non-motor features; furthermore, residualizing ΔLEDD from each post-DBS feature score prior to the PCA analysis did not change the outcomes when compared to the main analysis. However, as ΔLEDD is measured post-operatively and may itself reflect the DBS response, this analysis is merely addressing the confounding effect of ΔLEDD, not DBS-vs-medication attribution.

Finally, it is arguable that the outcomes of our analysis could merely be the result of natural disease progression. Among the most important features of advanced PD are treatment-resistant axial motor symptoms, and dementia. It is, however, unlikely that the observed interactions were only the results of the disease progression, as we excluded patients with dementia or major psychiatric symptoms at baseline from our dataset. In addition, we used the disease duration at baseline as a covariate in the moderation analysis to address this concern, including it reduces the likelihood that the associations are explained solely by baseline disease chronicity.

### Future outlook and clinical implications

These findings have direct implications for patient selection and post-operative management. Baseline mood assessment should be incorporated into DBS candidacy evaluation to identify patients states and identify those with higher risk of suboptimal gait and mobility recovery. Post-operative mood—particularly anxiety—monitoring and targeted intervention, including rehabilitation approaches combining motor and psychological components, such as dual-task training, may offer additional motor benefit, though prospective trials are needed. Future work should examine these interactions at the brain-network level to identify stimulation targets that address both motor and non-motor burden simultaneously.

### Limitations

Several limitations warrant consideration. First, the retrospective design and strict inclusion criteria reduced the available sample to 100 of 180 screened patients, potentially introducing selection bias toward more completely assessed cases. Second, the long-term follow-up sample (*n* = 33 at 3–5 years) is modest, though comparable to similar STN–DBS studies reporting 15–50 participants at equivalent timepoints [69]. Third, L-dopa dosage was not directly modeled to avoid overfitting; however, assessments conducted exclusively in MED–OFF state isolate DBS effects, and disease duration was included as a covariate in moderation models. Fourth, comparing PCA loadings across timepoints carries inherent mathematical limitations; we addressed this through consistent pre-processing and by analyzing projected follow-up scores on baseline axis and using mixed-effect models to track patients’ trajectories. In addition, repeated cognitive testing can introduce practice effects, although these are attenuated by the sparse assessment intervals; where present they bias serial scores toward apparent improvement, so the observed cognitive declines are conservative, while the MCI-subgroup improvement trend should be interpreted cautiously. In addition, the subgroup available at 3–5 years was broadly representative of the full cohort but was enriched for higher baseline motor severity; because baseline severity constrains the scope for subsequent change, the long-term motor findings may be modestly affected by this selection and warrant confirmation in larger, more completely followed cohorts. Finally, external validation for the clusters and cross-domain associations was not possible: no independent cohort meeting the inclusion criteria (complete item-level pre- and post-operative assessments across motor, cognitive, and mood domains) could be identified, reflecting a broader scarcity of harmonized multi-domain longitudinal DBS datasets. Prospective multicentre studies with pre-agreed assessment protocols will be essential to replicate these findings. Nonetheless, the current study deepens our understanding of the complicated behavioral features’ interactions following STN–DBS and it represents a crucial first step to plan further follow-up studies to investigate the complex interplay between motor and NMS following DBS surgery.

## Conclusion

This study suggests that STN–DBS outcomes in PD are not limited to global motor improvement and may be shaped by relationships spanning the motor, cognitive and mood domains. A core triad of gait, mobility, and FoG consistently dominated between-patient clinical variance across all timepoints, persisting as the primary source of unmet therapeutic need. In exploratory analyses, motor, cognitive, and mood features remained inter-related following STN–DBS, and baseline non-motor burden, particularly mood, may help explain heterogeneity in subsequent gait and mobility outcomes. These results support a broader, multi-domain view of pre-operative assessment, in which mood and cognitive evaluation may add value to candidacy discussions and represent candidate targets for future studies aiming to optimize motor recovery, rather than established determinants of outcome.

## Supplementary Information

Below is the link to the electronic supplementary material.

Supplementary file1 (DOCX 2159 kb)

## Data Availability

Anonymized data supporting the findings of this study are available from the corresponding author upon reasonable request.

## Code availability

The analysis code is available from the corresponding author upon reasonable request.
