# Supplementary material for: Non-motor symptoms burden and motor recovery following STN–DBS in Parkinson's disease: a data-driven longitudinal analysis
Source: J Neurol. 2026 Oct 1;273(10):636. doi: 10.1007/s00415-026-14160-x (PMC13630839; doi:10.1007/s00415-026-14160-x)
Supplement: Supplementary file 1 [file 415_2026_14160_MOESM1_ESM.docx]

**Non-Motor Symptoms Burden and Motor Recovery Following STN-DBS in Parkinson's Disease: A Data-Driven Longitudinal Analysis**

Journal of Neurology

Ghadir Abbas, Robert Peach, Uchralt Temuulen, Yunyou Tang, Tanmoy Sil, Anna Kufner, Matthias Endres, Jens Volkmann, Florian Lange, Martin M. Reich

Corresponding author: Prof. Dr. med. Martin M. Reich, Neurologische Klinik und Poliklinik, Universitätsklinikum Würzburg, Josef-Schneider-Straße 11, Haus B1, 97080 Würzburg, Germany. [reich_m1@ukw.de](mailto:reich_m1@ukw.de)

# **Supplementary Methods:**

**Participants:**
Patients with PD who underwent STN-DBS surgery at the Department of Neurology, University Hospital Würzburg, Germany, between 2010 and 2020 were included. Inclusion criteria were: (1) confirmed diagnosis of PD according to the guidelines of the Deutsche Gesellschaft für Neurologie (DGN); (2) availability of complete pre-operative clinical assessments across all three domains; and (3) at least one post-operative follow-up visit at approximately 12 months. Patients were excluded if they had dementia or a major psychiatric disorder at baseline, lacked full neurobehavioral assessment before DBS, or had inconsistent outcome measures across timepoints. All participants provided written informed consent. The study was approved by the Ethics Committee of the University of Würzburg (248/20-me) in accordance with the Declaration of Helsinki.

**Clinical Assessment:**

Data were retrospectively collected from three timepoints: baseline (pre-DBS), 1-year post-DBS follow-up (1-y FU), and 3–5-year post-DBS follow-up (3–5-y FU). LEDD was calculated at pre-DBS and 1-y FU using established conversion factors [1]. Motor function was assessed using MDS-UPDRS Parts II and III. Assessments were performed in the MED-OFF state (after overnight withdrawal of dopaminergic medication) before DBS, and in the MED-OFF / STIM-ON state at the 1-year and 3–5-year follow-ups [2]. Cognitive function was assessed with MoCA [3] or MDRS [4]; the scale used remained consistent within each patient across all timepoints. Mood was assessed with the HDRS [5]. For MDS-UPDRS II and III, paired t-tests were used as the primary test. For scales with known ceiling or floor effects (MoCA, MDRS, HDRS), Wilcoxon signed-rank tests were used (α = 0.05). All p-values were corrected for multiple comparisons using the Benjamini-Hochberg FDR (FDR_BH) procedure applied across all five comparisons simultaneously.

**Clustering Analysis**:

Clustering was performed on 22 clinical sub-domain scores spanning motor (speech, facial expression, rigidity, bradykinesia, mobility, gait, freezing of gait, postural instability, posture, tremor), mood (affect, anxiety, somatic symptoms, sleep, cognitive aspects, psychomotor function), and cognitive (attention, language, memory, orientation) domains. Cognitive sub-domain scores were derived from MoCA sub-scores, with Mattis sub-scores rescaled to the MoCA range and inverted so that higher values indicate greater impairment. Missing baseline values were imputed by multiple imputation by chained equations (MICE; scikit-learn IterativeImputer, round-robin conditional regression with a BayesianRidge estimator, median initialisation, ≤ 25 iterations, converged in 4; seed 147). The imputation model was fitted on pre-DBS data only (n = 100) across 21 numeric clinical features - each imputed conditionally from all others - and applied to post-DBS data by transformation to prevent cross-timepoint leakage; sex (never missing) was excluded from the round-robin and reappended afterwards. The primary clustering used a single completed dataset; to verify robustness to imputation uncertainty, 20 multiply-imputed datasets (sample_posterior=True; seeds 10147–10166) were each carried independently through the identical standardisation–PCA–k-means pipeline, with partitions aligned to the primary solution by Hungarian matching and agreement quantified by the Adjusted Rand Index and per-patient assignment stability. Features were then z-score standardised with a scaler also fit on pre-DBS data. PCA (sklearn.decomposition.PCA, Python 3.11) was applied to the standarised pre-DBS data, retaining components explaining 99.9% of cumulative variance. The optimal number of clusters was determined by evaluating silhouette scores for k = 2–10, with k-means clustering (20 initialisations) applied to the PCA-embedded data. Cluster stability was assessed by non-parametric bootstrap resampling (B = 500), quantifying the reproducibility of each cluster as the mean cluster-wise Jaccard similarity between the original partition and partitions obtained on resampled data (Hennig 2007); following that criterion, clusters with a mean Jaccard ≥ 0.85 were considered stable, 0.60–0.85 moderately stable, and < 0.60 dissolved. This procedure was repeated for k = 2–10 to confirm the value of k selected by the silhouette score. Clusters were labelled such that Cluster 1 consistently denotes the higher-impairment group. To assess temporal stability, post-DBS patients were projected into the pre-DBS PCA space using the pre-DBS transformation and assigned to the nearest centroid without refitting the model. Transition matrices were computed for patients with data at both timepoints. Between-cluster differences were assessed using independent-samples t-tests for normally distributed features (Shapiro–Wilk, p > 0.05) and Mann–Whitney U tests otherwise.

**Dominant patterns of clinical covariation**:

PCA was performed on 13 sub-domain scores (Online Resources; Table S1). Tremor items were excluded because tremor correlates poorly with other motor features and can dominate early PCs, masking clinically relevant gait- and mobility-related variance [6]. Bradykinesia asymmetry score (BAS) and rigidity asymmetry score (RAS) were calculated as the absolute difference between left- and right-sided MDS-UPDRS Part III sub-scores quantifying the magnitude (not the direction) of motor asymmetry [7]. Cognitive sub-domain scores were inverted so that higher values uniformly indicate greater impairment. Missing values were imputed only for features with <15% missingness using median-initialised expectation–maximisation (EM) to preserve principal-component structure. Sub-domain scores were then min–max normalised prior to the PCA. Cross-sectional PCAs were fitted independently at pre-DBS, 1-y FU, and 3–5-y FU. Structural similarity of PCA loadings across timepoints was quantified using Procrustes analysis. For per-feature longitudinal comparisons, the normality of the paired pre-DBS-to-follow-up differences was assessed with the Shapiro–Wilk test; a Wilcoxon signed-rank test was used where the differences departed from normality and a paired t-test otherwise, with Benjamini–Hochberg FDR correction. To quantify within-patient longitudinal change on fixed clinical dimensions, the PCA was fitted on pre-DBS data only; the 1-year and 3–5-year observations were projected onto the fixed baseline loadings. Change in the projected component scores relative to baseline was tested with linear mixed-effects models (random intercept per patient; timepoint as a fixed effect, baseline as reference), across 234 patient-visits (n = 100, 100, and 33 at pre-DBS, 1-year, and 3–5-year follow-up). The independent per-timepoint PCAs were retained as a secondary analysis of covariance-structure change (Procrustes disparity restricted to the first five principal components, against a permutation null, n_perm = 5000). To assess whether DBS-induced reductions in LEDD confounded post-operative PCA structure, ΔLEDD was regressed out of each post-DBS clinical sub-domain score using simple linear regression and the PCA was repeated on the residuals. Structural similarity between the original and LEDD-residualised solutions was quantified by Procrustes analysis. Spearman correlations between ΔLEDD and each clinical change score were computed and FDR-corrected.

**Moderation Analysis**:

# Moderation was tested using analysis of covariance (ANCOVA) rather than change-score models, to avoid the mathematical coupling between a change score and its own baseline [ref]. For each motor outcome, the 1-year follow-up score was regressed on (a) the pre-DBS baseline value of that outcome, (b) a non-motor moderator, (c) the baseline × moderator interaction, and (d) age and disease duration at surgery as covariates. A statistically significant interaction term was taken as evidence that the moderator modified the degree of post-DBS recovery. All continuous predictors were min–max normalised to [0, 1]; both constituents of each interaction were mean-centred before the product term was formed, and the interaction term was not re-standardised, so reported coefficients are on the common [0, 1] range-normalised scale (not z-standardised). Baseline (pre-DBS) non-motor severity was the primary moderator; 1-year follow-up non-motor severity was examined separately as a concurrent cross-domain association, because it is measured at the same visit as the outcome and cannot establish temporal precedence. Motor outcomes comprised mobility, gait, and freezing of gait, and a composite motor score; two composite non-motor domains (cognition and mood) were additionally tested against the composite motor outcome. In total, 68 models were fitted (32 with baseline moderators, 32 with post-DBS moderators, and 4 composite-domain models). For each motor outcome at each timepoint, interaction p-values were corrected across the eight candidate non-motor moderators using the Benjamini–Hochberg FDR procedure. All models were fitted with HC3 heteroscedasticity-consistent robust standard errors, and regression diagnostics (Ramsey RESET, Breusch–Pagan, Cook's distance, and variance inflation factors).

# **Supplementary Tables:**

| **Table S1: Item content of each domain’s subscales.** | | | | | |
| --- | --- | --- | --- | --- | --- |
| **Motor domain** | | **Cognitive domain** | | **Mood domain** | |
| Mobility | MDS-UPDRS-II: 2, 11  MDS-UPDRS-III: 9 | Attention | MoCA: Attention  Mattis: I A-D, V D1-2, VI E1-F1 | Affect: | HDRS: 1, 2 |
| Gait | MDS-UPDRS-II: 12  MDS-UPDRS-III: 10, 12 | Language: | MoCA: Naming, Language  Mattis: II A1-A4 | Anxiety: | HDRS: 10, 11 |
| FoG | MDS-UPDRS-II: 13  MDS-UPDRS-III: 11 | Memory: | MoCA: Memory  Mattis: VI A2-B2, E2-F2 | Psychosomatic: | HDRS: 12, 13 |
| BAS | MDS-UPDRS-III: 4, 5, 6, 7, 8 | Orientation: | MoCA: Orientation  Mattis: V C | Psychomotor: | HDRS: 8, 9 |
| RAS | MDS-UPDRS-III: 3 |  |  |  |  |

Table S1: Item content of each domain's subscales. Mapping of individual MDS-UPDRS-II, MDS-UPDRS-III, MoCA, Mattis and HDRS items to the motor, cognitive and mood sub-domain scores used in all analyses. FoG freezing of gait, BAS bradykinesia asymmetry score, RAS rigidity asymmetry score

| **Table S2: Demographic and clinical summary.** | | | | | | | | | |
| --- | --- | --- | --- | --- | --- | --- | --- | --- | --- |
|  | **Pre-DBS** | | | **1-y FU** | | | **3-5-y FU** | | |
|  | **N** | **Mean** | **SD** | **N** | **Mean** | **SD** | **N** | **Mean** | **SD** |
| Age at surgery (years) | 100 | 60.278 | 7.620 |  |  |  |  |  |  |
| Disease duration at surgery (years) | 100 | 11.100 | 4.347 |  |  |  |  |  |  |
| LEDD (mg/day) | 100 | 1070 | 497 | 99 | 509 | 358 |  |  |  |
| Sum MDS-UPDRS II | 94 | 12.894 | 7.150 | 92 | 9.967 | 7.576 | 33 | 10.15 | 7.7 |
| Sum MDS-UPDRS III | 100 | 43.530 | 12.010 | 100 | 21.810 | 10.541 | 35 | 26.34 | 14.74 |
| Sum Mattis | 44 | 141.386 | 2.365 | 44 | 139.545 | 4.567 | 4 | 138 | 6 |
| Sum MoCA | 56 | 26.786 | 1.951 | 56 | 26.500 | 3.191 | 30 | 24.46 | 3.9 |
| Sum HDRS | 100 | 4.740 | 4.049 | 98 | 4.061 | 3.858 | 34 | 5.8 | 5 |

Table S2: Demographic and clinical summary. Number of available observations, mean and standard deviation for age at surgery, disease duration, LEDD and summed clinical scale scores at pre-DBS, 1-year and 3–5-year follow-up. LEDD levodopa equivalent daily dose.

| **Table S3: Per-patient subthalamic stimulation parameters at one-year and long-term (3–5-year) follow-up.** | | | | | | | | | | | | | | | | | | | | | | | | | | | | | |
| --- | --- | --- | --- | --- | --- | --- | --- | --- | --- | --- | --- | --- | --- | --- | --- | --- | --- | --- | --- | --- | --- | --- | --- | --- | --- | --- | --- | --- | --- |
| **Pat.** | **Sex** | **Age [y]** | **Dis. dur. [y]** | **Hardware** | | **STN right — 1-year** | | | | | **STN left — 1-year** | | | | | | **STN right — 3–5-year** | | | | | | **STN left — 3–5-year** | | | | | **LEDD pre** | **LEDD post** |
|  |  |  |  | **Firm** | **Stimulator** | **+** | **−** | **mA** | **µs** | **Hz** | **+** | **−** | **mA** | **µs** | **Hz** | **+** | | **−** | **mA** | **µs** | **Hz** | **+** | | **−** | **mA** | **µs** | **Hz** |  |  |
| 2 | m | 53.8 | 13 | Medtronic | Activa PC | G | 2 | 6 | 60 | 130 | G | 1 | 4 | 60 | 130 | G | | 0 | 6.5 | 60 | 80 | G | | 2 and 3 | 5 | 60 | 80 | 1915 | 235 |
| 3 | m | 60.3 | 13 | Medtronic | Activa PC | G | 3 | 1.8 | 60 | 130 | G | 2 | 2.6 | 60 | 130 |  | |  |  |  |  |  | |  |  |  |  | 1940 | 0 |
| 4 | m | 57.6 | 20 | Medtronic | Activa PC | G | 1 | 4.5 | 60 | 90 | G | 1 | 4.5 | 60 | 90 | G | | 1 | 4 | 60 | 90 | G | | 1 | 3 | 60 | 90 | 2660 | 310 |
| 6 | f | 59.4 | 15 | Medtronic | Activa PC | G | 2 | 2.9 | 60 | 140 | G | 1 | 3.1 | 60 | 140 | G | | 1 |  | 60 | 90 | G | | 1 | 4 | 60 | 90 | 1330 | 550 |
| 8 | m | 55.8 | 6 | Medtronic | Activa PC | G | 2 | 2 | 60 | 130 | G | 2 | 4.5 | 60 | 160 | G | | 1,2 | 2.5 | 60 | 130 | G | | 1 | 4.5 | 60 | 130 | 1440 | 905 |
| 11 | m | 57 | 12 | BostonSci | Vecrise | G | 3 | 5.2 | 40 | 130 | G | 2 | 3.5 | 40 | 130 | G//G | | 1 and 4 //4 | 8 //4.5 | 60 | 74 | G | | 3 and 4 | 4.5 | 60 | 74 | 1000 | 475 |
| 14 | f | 47.9 | 10 | Medtronic | Activa PC | G | 2 | 2.5 | 60 | 130 | G | 2 | 2.3 | 60 | 180 | G | | 2 | 2.8 | 60 | 120 | G | | 2 | 3.8 | 60 | 120 | 630 | 0 |
| 15 | f | 62 | 12 | BostonSci | Vecrise | G | 2(50%) 3(50%) | 4 | 40 | 174 | G | 2 | 3.4 | 40 | 130 | G | | 3 (50%) and 4 (50%) | 4.2 | 40 | 174 | G | | 3 (75%) and 4 (25%) | 5 | 40 | 174 | 275 | 290 |
| 16 | m | 70.1 | 13 | Medtronic | Activa PC | G | 2 | 3.5 | 60 | 130 | G | 2 | 3.3 | 60 | 130 | G | | 1 | 4 | 60 | 125 | G | | 2 | 3.3 | 60 | 125 | 1400 | 1200 |
| 17 | f | 63.3 | 12 | Medtronic | Activa PC | G | 2 | 3.5 | 60 | 130 | G | 2 | 4 | 60 | 130 | G | | 2 and 3 | 3.2 | 60 | 180 | G | | 2 and 3 | 2.1 | 60 | 180 | 1290 | 915 |
| 18 | f | 59.4 | 15 | Medtronic | Activa PC | G | 2 | 1.6 | 60 | 180 | G | 2 | 4.3 | 60 | 180 | G | | 4 | 2.9 | 60 | 179 | G | | 3 | 4.4 | 40 | 179 | 295 | 155 |
| 19 | m | 60 | 7 | BostonSci | Vecrise | G | 3 | 3 | 20 | 174 | G | 2 | 4 | 30 | 130 | 4 | | 3 | 3.6 | 30 | 130 | 4 | | 2 and 3 | 5.3 | 20 | 74 | 1230 |  |
| 20 | m | 60 | 12 | BostonSci | Vecrise | G | 2 | 3.5 | 60 | 185 | G | 6 | 2.9 | 60 | 185 | G | | 3 | 4 | 60 | 185 | G | | 3 | 3.6 | 60 | 185 | 1900 | 460 |
| 21 | m | 73 | 10 | BostonSci | Vecrise | G | 2 | 5 | 60 | 174 | G | 3(50%) 2(50%) | 4.3 | 60 | 174 | G | | 2 | 5.7 | 60 | 174 | G | | 2 (70%) and 3 (30%) | 5.4 | 40 | 174 | 1335 | 660 |
| 22 | f | 74.1 | 17 | Medtronic | Actica PC | G // G | 2 // 1 | 1,8 // 1,5 | 60 | 125 | G | 1 | 2.4 | 60 | 125 | G//G | | 2//2 | 2.2 // 1.7 | 60 | 125 | G | | 2 | 2.6 | 60 | 125 | 1133 | 415 |
| 23 | m | 47.4 | 23 | Medtronic | Activa PC | G | 1 | 3 | 60 | 160 | G | 1 | 3.5 | 60 | 160 | G | | 1 | 2.8 | 60 | 140 | G | | 2 | 5 | 60 | 140 | 740 | 0 |
| 24 | m | 58.8 | 6 | Medtronic | Activa PC | G | 3 | 3.1 | 60 | 130 | G | 2 and 3 | 2.2 | 60 | 130 | G | | 3 and 4 | 4.2 | 40 | 174 | G | | 3 and 4 | 4.2 | 40 | 208 | 1790 | 1790 |
| 25 | f | 66.1 | 14 | Medtronic | Activa PC | G | 2 | 2 | 60 | 125 | G | 2 // 3 | 4.1 // 2.2 | 60 | 125 |  | |  |  |  |  |  | |  |  |  |  | 500 | 300 |
| 26 | f | 58 | 11 | BostonSci | Vecrise | G | 3 | 4.5 | 60 | 174 | G | 2 | 4 | 60 | 174 | G | | 2 (70%) and 3 (30%) | 4.7 | 40 | 130 | G | | 2 | 4 | 60 | 174 | 715 | 310 |
| 27 | m | 64 | 5 | BostonSci | Vecrise | G | 4 | 4.8 | 60 | 130 | G | 3 | 3.1 | 60 | 130 | G | | 4 (75%) and 5 (25%) | 4.2 | 60 | 174 | G | | 2 (25%) and 3 (75%) | 4 | 40 | 174 | 725 | 575 |
| 28 | f | 55 | 13 | BostonSci | Vecrise | G | 2 | 3.8 | 60 | 170 | G | 3 | 3.7 | 60 | 170 | G | | 3 | 4 | 60 | 170 | G | | 4 | 4.2 | 60 | 170 | 1050 | 340 |
| 29 | m | 64.9 | 10 | Medtronic | Activa PC | G | 2 | 2.7 | 60 | 180 | G | 2 | 3.3 | 60 | 180 | G | | 1 and 2 | 3 | 40 | 179 | G | | 2 | 2.8 | 40 | 179 | 1475 | 800 |
| 30 | f | 55.6 | 6 | Medtronic | Activa PC | G | 3 | 2.2 | 60 | 130 | G | 2 | 3.1 | 60 | 130 | G | | 2 | 1.7 | 60 | 130 | G | | 1 | 1.6 | 60 | 130 | 2180 | 250 |
| 31 | m | 60.3 | 18 | Medtronic | Activa PC | G | 2 | 4.3 | 60 | 180 | G | 1 | 4 | 60 | 180 | G | | 1 and 2 | 4 | 90 | 90 | G | | 1 | 4.4 | 60 | 90 | 1550 | 800 |
| 32 | m | 66.1 | 14 | Medtronic | Activa PC | G | 1 | 3.6 | 60 | 130 | G | 1 | 2.8 | 60 | 130 |  | |  |  |  |  |  | |  |  |  |  | 1000 | 425 |
| 34 | f | 54 | 6 | BostonSci | Vecrise | G | 3 | 1.8 | 60 | 132 | G | 3 | 3 | 60 | 132 | G | | 4 | 3.5 | 60 | 136 | G | | 4 | 3.7 | 60 | 136 | 1360 | 725 |
| 35 | m | 50.5 | 11 | Medtronic | Activa PC | G | 1 | 2.4 | 60 | 130 | G | 2 | 3.5 | 60 | 130 | G | | 1 | 2.4 | 60 | 130 | G | | 2 | 4.5 | 60 | 130 | 1025 | 160 |
| 36 | m | 60.4 | 11 | Medtronic | Activa PC | G | 2 | 2 | 60 | 130 | G | 2 | 2.5 | 60 | 130 | G | | 2 | 2 | 60 | 130 | G | | 1 | 5.1 | 60 | 130 | 725 | 100 |
| 37 | m | 54.8 | 19 | Medtronic | Activa PC | G | 2 | 3.5 | 60 | 130 | G | 1 | 4.3 | 60 | 130 | G | | 3 | 2.5 | 60 | 179 | G | | 3 | 3.1 | 60 | 179 | 1570 | 400 |
| 38 | m | 50 | 12 | BostonSci | Vecrise | G | 4-(50%) 5-(50%) | 4.5 | 60 | 174 | G | 3 | 3.7 | 60 | 174 | G | | 3 and 4 | 5.1 | 60 | 147 | G | | 3 | 4.1 | 60 | 147 | 1550 | 660 |
| 39 | m | 54.4 | 6 | Medtronic | Activa PC | G | 2 | 2 | 60 | 180 | G | 3 | 4 | 60 | 180 |  | |  |  |  |  |  | |  |  |  |  | 1885 | 775 |
| 40 | m | 55.6 | 10 | Medtronic | Activa PC | G | 1 | 3 | 60 | 160 | G | 0 | 4 | 60 | 160 | G | | 2 | 3.6 | 60 | 180 | G | | 2 | 4.7 | 60 | 180 | 1260 | 690 |
| 41 | f | 52.7 | 11 | Medtronic | Activa PC | G | 1 and 2 | 3 | 60 | 130 | G | 1 and 2 | 2.5 | 60 | 130 | G | | 2 and 3 | 3.3 | 60 | 210 | G | | 2 and 3 | 3.7 | 60 | 210 | 1070 | 765 |
| 42 | m | 73.3 | 18 | Medtronic | Activa PC | G | 2 | 3.5 | 60 | 180 | G | 1 | 2.5 | 60 | 180 |  | |  |  |  |  |  | |  |  |  |  | 2050 | 600 |
| 43 | f | 68.3 | 6 | Medtronic | Activa PC | G | 1 | 1.5 | 60 | 180 | 2 | 1 | 3 | 60 | 180 |  | |  |  |  |  |  | |  |  |  |  | 725 | 315 |
| 44 | m | 48.8 | 7 | BostonSci | Vecrise | G | 3 | 2.5 | 60 | 174 | G | 2 | 2.5 | 60 | 174 | G | | 2 (25%) and 3 (75%) | 3 | 50 | 149 | 8 | | 2 and 3 | 3.7 | 30 | 149 | 310 | 485 |
| 45 | m | 71.4 | 9 | Medtronic | Activa PC | G | 1 | 3 | 60 | 180 | G | 1 and 2 | 3 | 60 | 180 | G | | 2 | 2.5 | 60 | 180 | G | | 0 | 2.4 | 60 | 180 | 140 | 0 |
| 46 | m | 48.9 | 7 | BostonSci | Vecrise | G | 6 | 3.5 | 60 | 179 | G | 4 | 3.5 | 60 | 179 | G | | 4 | 4.5 | 60 | 179 | G | | 3 | 4 | 60 | 179 | 800 | 0 |
| 47 | m | 66.7 | 8 | BostonSci | Vecrise | G | 2 | 3.5 | 30 | 130 | G | 1 | 5.5 | 20 | 174 | G | | 2 and 3 | 3.6 | 50 | 185 | G | | 2 (10%), 3 (10%) , 4(10%), 5 (24%), 6 (23%), 7 (23%) | 2.6 | 30 | 185 | 1580 | 660 |
| 48 | m | 52.6 | 9 | BostonSci | Vecrise | G | 3 | 4.8 | 60 | 174 | G | 6 | 5.6 | 60 | 174 |  | |  |  |  |  |  | |  |  |  |  | 1610 | 500 |
| 50 | m | 61.6 | 11 | Medtronic | Activa PC | G | 2 | 3 | 60 | 180 | G | 2 and 3 | 3.6 | 60 | 180 |  | |  |  |  |  |  | |  |  |  |  | 775 | 105 |
| 51 | m | 67.9 | 11 | Medtronic | Activa PC | 3 | 2 | 6.3 | 60 | 160 | 2 | 1 | 5.3 | 60 | 160 |  | |  |  |  |  |  | |  |  |  |  | 1670 | 1130 |
| 52 | m | 46.7 | 13 | Medtronic | Activa PC | G | 2 | 4.5 | 60 | 130 | G | 2 | 3.2 | 60 | 130 |  | |  |  |  |  |  | |  |  |  |  | 1165 | 600 |
| 55 | m | 53.1 | 8 | BostonSci | Vecrise | G | 3 | 3 | 60 | 128 | G | 3 | 2.8 | 60 | 128 |  | |  |  |  |  |  | |  |  |  |  | 1065 | 220 |
| 74 | f | 57.2 | 13 | Medtronic | Activa PC | G | 1 | 1.5 | 60 | 130 | G | 2 | 2.5 | 60 | 130 |  | |  |  |  |  |  | |  |  |  |  | 610 | 310 |
| 78 | m | 56.2 | 5 | BostonSci | Versice PC + Cartesia | G | 5(25%),6(25%),8(50%) | 2.7 | 60 | 179 | G | 1 | 2.2 | 60 | 179 |  | |  |  |  |  |  | |  |  |  |  | 560 | 600 |
| 80 | m | 67.4 | 10 | BostonSci | Versice PC | G | 4(80%),5(20%) | 2.9 | 60 | 174 | G | 3(30%),4(70%) | 2.9 | 60 | 174 |  | |  |  |  |  |  | |  |  |  |  | 500 | 360 |
| 81 | m | 68.2 | 14 | BostonSci | Versice PC + Cartesia | G | 8 | 1.6 | 60 | 130 | G | 8 | 3.2 | 60 | 130 |  | |  |  |  |  |  | |  |  |  |  | 1660 | 615 |
| 82 | m | 68.2 | 17 | Medtronic | Activa PC | G | 2 | 2 | 60 | 130 | G | 3 | 2.4 | 60 | 130 |  | |  |  |  |  |  | |  |  |  |  | 950 | 680 |
| 83 | f | 60.9 | 14 | Medtronic | Activa PC | G | 2,3 (50% each) | 3.5 | 60 | 180 | G | 2 | 2.5 | 60 | 180 |  | |  |  |  |  |  | |  |  |  |  | 530 | 155 |
| 84 | f | 58.8 | 9 | BostonSci | Versice PC + Cartesia | G | 8 | 3.8 | 60 | 130 | G | 5 (10%), 6 (10%), 7(10%), 8(70%) | 3.4 | 60 | 130 |  | |  |  |  |  |  | |  |  |  |  | 770 | 280 |
| 85 | f | 67.2 | 8 | BostonSci | Versice Rechargeable | G | 3 | 1.8 | 60 | 180 | G | 2,3(50% each) | 2 | 60 | 130 |  | |  |  |  |  |  | |  |  |  |  | 875 | 495 |
| 86 | m | 62.9 | 9 | BostonSci | Versice PC + Cartesia | G | 8 | 0.5 | 60 | 179 | G | 8 | 0.7 | 60 | 179 |  | |  |  |  |  |  | |  |  |  |  | 1110 | 1050 |
| 87 | m | 72.7 | 10 | BostonSci | Versice PC + Cartesia | G | 2-4(Ringmode) | 1.7 | 60 | 130 | G | 5-7(Ringmode) | 2 | 60 | 130 |  | |  |  |  |  |  | |  |  |  |  | 870 | 720 |
| 88 | m | 69.7 | 12 | BostonSci | Versice PC + Cartesia | G | 2(50%),3(50%) | 2.5 | 60 | 130 | G | 2,3,4(50%), 5,6,7(50%) | 2.4 | 60 | 130 |  | |  |  |  |  |  | |  |  |  |  | 1640 | 665 |
| 90 | f | 67.1 | 23 | BostonSci | Vecrise PC | G | 5(4%),6(3%),7(3%),8(90%) | 3.3 | 60 | 170 | G | 5(25%),6(75%) | 1 | 60 | 128 |  | |  |  |  |  |  | |  |  |  |  | 1165 | 735 |
| 91 | m | 50.7 | 10 | BostonSci | Vecrise Gevia + Cartesia | G | 2 (25%), 3(75%) | 2.1 | 60 | 130 | G | 7 | 2.4 | 60 | 130 |  | |  |  |  |  |  | |  |  |  |  | 550 | 0 |
| 92 | f | 78.0 | 10 | BostonSci | Vecrise Gevia | G | 5(50%),6(50%) | 3.8 | 60 | 130 | G | 5(38%),7(12%),8(50%) | 2 | 60 | 130 |  | |  |  |  |  |  | |  |  |  |  | 590 | 400 |
| 93 | m | 65.2 | 12 | BostonSci | Versice PC + Cartesia | G | 5(20%),6(20%),7(60%) | 3.3 | 60 | 130 | G | 7 | 3.3 | 60 | 130 |  | |  |  |  |  |  | |  |  |  |  | 1520 | 475 |
| 94 |  | 70.4 | 23 | BostonSci | Versice PC + Cartesia | G | 2(70%),5(30%) | 3.2 | 60 | 130 | G | 5-7(Rm) | 3.2 | 60 | 130 |  | |  |  |  |  |  | |  |  |  |  | 655 | 475 |
| 95 | f | 61.9 | 6 | BostonSci | Gevia | G | 6(15%), 7(15%), 8(70%) | 2 | 60 | 130 | G | 7, 8 (50% each) | 1.5 | 60 | 130 |  | |  |  |  |  |  | |  |  |  |  | 690 | 280 |
| 96 | f | 64.6 | 13 | BostonSci | Vecrise Gevia | G | 7 | 1.2 | 60 | 130 | G | 6 | 1.2 | 60 | 130 |  | |  |  |  |  |  | |  |  |  |  | 960 | 1800 |
| 97 | f | 63.0 | 14 | BostonSci | Versice PC + Cartesia | G | 5-7(Rm 80%), 8(20%) | 2 | 60 | 130 | G | 5-7(Rm 50%), 8 (50%) | 1.5 | 60 | 130 |  | |  |  |  |  |  | |  |  |  |  | 870 | 675 |
| 98 | f | 64 | 10 | BostonSci | Vecrise Gevia | G | 2(38%),3(12%),5(38%),6(12%) | 1.8 | 60 | 130 | G | 6(100%) | 2.4 | 60 | 130 |  | |  |  |  |  |  | |  |  |  |  | 655 | 550 |
| 99 | m | 54.2 | 10 | BostonSci | Vecrise PC | G | 7 | 3.8 | 60 | 130 | G | 5 | 1.8 | 60 | 130 |  | |  |  |  |  |  | |  |  |  |  | 1200 | 680 |
| 100 | m | 68 | 19 | BostonSci | Vecrise PC | G | 2(80%),5(20%) | 7.2 | 40 | 79 | G | 5 | 5.8 | 30 | 79 |  | |  |  |  |  |  | |  |  |  |  | 1280 | 1000 |
| 101 | m | 54.3 | 10 | BostonSci | Gevia | G | 5(30%), 8(70%) | 3.9 | 60 | 185 | G | 6,7 (50% each) | 2.8 | 60 | 132 |  | |  |  |  |  |  | |  |  |  |  | 255 | 0 |
| 102 | m | 56.8 | 5 | BostonSci | Gevia | G | 8 | 2.2 | 60 | 130 | G(50%),5 (25%), 7(25%) | 6(20%), 8(80%) | 5.2 | 60 | 130 |  | |  |  |  |  |  | |  |  |  |  | 400 | 100 |
| 103 | f | 56 | 8 | BostonSci | Vecrise Gevia | G | 5(50%),6(50%) | 4 | 50 | 179 | G | 5(40%),6(40%),8(20%) | 2.2 | 40 | 149 |  | |  |  |  |  |  | |  |  |  |  | 1025 | 655 |
| 104 | m | 69 | 9 | BostonSci | Vecrise Gevia | G | 4(100%) | 3 | 60 | 130 | G | 2(30%),3(30%),5(20%),6(20%) | 3.7 | 60 | 130 |  | |  |  |  |  |  | |  |  |  |  | 1490 | 540 |
| 105 | m | 52.2 | 12 | BostonSci | Vecrise Gevia | G | 8 | 2.8 | 40 | 130 | G | 8 | 4 | 40 | 130 |  | |  |  |  |  |  | |  |  |  |  | 1600 | 1140 |
| 106 | m | 58.2 | 8 | BostonSci | Vecrise Gevia | G | 8 | 2.7 | 60 | 130 | G | 6(25%),7(25%),8(50%) | 2.8 | 60 | 130 |  | |  |  |  |  |  | |  |  |  |  | 980 | 530 |
| 107 | f | 57 | 18 | BostonSci | Vecrise PC | G | 2(34%), 3(33%),4(33%) | 2.7 | 60 | 179 | G | 8(100%) | 2.5 | 70 | 238 |  | |  |  |  |  |  | |  |  |  |  | 1230 | 500 |
| 108 | f | 50 | 6 | BostonSci | Vecrise Gevia | G | 6(100%) | 2.6 | 60 | 130 | G | 3(30%),4(10%),6(45%),7(15%) | 1.7 | 60 | 130 |  | |  |  |  |  |  | |  |  |  |  | 760 | 320 |
| 109 | f | 65.8 | 11 | BostonSci | Versice PC + Cartesia | G | 3,4 (35%each), 8(30%) | 3.5 | 60 | 185 | G | 7 | 1.7 | 40 | 185 |  | |  |  |  |  |  | |  |  |  |  | 1185 | 425 |
| 110 | m | 59.1 | 14 | BostonSci | Versice PC + Cartesia | G | 2,3,4 (33%) | 3.3 | 30 | 132 | G | 8 | 2.9 | 60 | 132 |  | |  |  |  |  |  | |  |  |  |  | 1220 | 1390 |
| 111 | f | 67 | 12 | BostonSci | Vecrise PC | G | 8 | 3 | 60 | 130 | G | 7 | 3 | 60 | 130 |  | |  |  |  |  |  | |  |  |  |  | 895 | 225 |
| 112 | m | 53.1 | 5 | BostonSci | cartesia Gevia | G | 5,6,7,(20% each), 8(40%) | 2 | 60 | 179 | G | 6 | 2.7 | 60 | 179 |  | |  |  |  |  |  | |  |  |  |  | 75 | 0 |
| 114 | m | 58.1 | 7 | BostonSci | cartesia | G | 7(70%), 8(30%) | 2.2 | 60 | 130 | G | 3,6 (50% each) | 2.4 | 60 | 130 |  | |  |  |  |  |  | |  |  |  |  | 900 | 500 |
| 115 | m | 51.0 | 9 | BostonSci | cartesia Gevia | G | 2,3,4,(Rm) | 3.1 | 60 | 130 | G | 2(40%),5(60%) | 3.6 | 60 | 130 |  | |  |  |  |  |  | |  |  |  |  | 385 | 315 |
| 116 | m | 64 | 14 | BostonSci | Vecrise Gevia | G | 5(50%),7(50%) | 2.5 | 60 | 130 | G | 3(100%) | 2.5 | 60 | 130 |  | |  |  |  |  |  | |  |  |  |  | 1215 | 755 |
| 117 | m | 42 | 8 | BostonSci | Vecrise Gevia | G | 3(60%),6(40%) | 4.4 | 60 | 185 | G | 7(100%) | 1.5 | 60 | 132 |  | |  |  |  |  |  | |  |  |  |  | 400 | 255 |
| 118 | m | 74.0 | 10 | BostonSci | Vecrise Gevia | G | 2,3,4(27% each), 5,6,7(7%) | 2.3 | 30 | 130 | G | 2(22%),4(68%),5(2%),7(8%) | 3.8 | 60 | 130 |  | |  |  |  |  |  | |  |  |  |  | 1300 | 940 |
| 120 | m | 46.2 | 9 | BostonSci | Vecrise Gevia | G | 5 | 1.9 | 60 | 179 | G | 7 | 1.1 | 60 | 179 |  | |  |  |  |  |  | |  |  |  |  | 785 | 475 |
| 121 | m | 55.8 | 3 | BostonSci | Vecrise Gevia | G | 5-7(RM) | 1.6 | 60 | 223 | G | 5(25%), 7(75%) | 4.8 | 60 | 223 |  | |  |  |  |  |  | |  |  |  |  | 0 | 0 |
| 122 | m | 62 | 11 | BostonSci | Vecrise Gevia Rechargable | G | 2(50%),4(50%) | 3 | 60 | 130 | G | 5(50%),6(50%) | 2.2 | 60 | 130 |  | |  |  |  |  |  | |  |  |  |  | 870 | 1000 |
| 123 | m | 49.8 | 8 | BostonSci | Vecrise Gevia Cartesia | G | 2,3 (50%) | 3 | 60 | 179 | G | 2,4 (50%) | 3.6 | 60 | 179 |  | |  |  |  |  |  | |  |  |  |  | 1410 | 530 |
| 124 | m | 71.3 | 7 | BostonSci | Vecrise Gevia Cartesia | G | 7(60%), 4(40%) | 3.9 | 60 | 179 | G | 7,4 (50% each) | 3.9 | 60 | 179 |  | |  |  |  |  |  | |  |  |  |  | 975 | 425 |
| 125 | m | 51.9 | 8 | BostonSci | Genus R16 | G | 2(75%),4(25%) | 3,3 | 60 | 185 | G | 1,2 (50%each) | 3.1 | 60 | 185 |  | |  |  |  |  |  | |  |  |  |  | 1135 | 400 |
| 126 | m | 63.3 | 8 | Medtronic | Percept | G | 2b | 3 | 60 | 135 | G | 1a-c | 4.5 | 60 | 180 |  | |  |  |  |  |  | |  |  |  |  | 1470 | 605 |
| 127 | m | 54.7 | 9 | BostonSci | Genus R16 | G | 2(6%), 3(4%), 4(7%), 5(26%), 6(27%), 7(27%) | 2.5 | 60 | 185 | G | 2(24%), 3(23%), 4(23%), 5(10%), 6(10%), 7(10%) | 2.5 | 60 | 139 |  | |  |  |  |  |  | |  |  |  |  | 930 | 300 |
| 128 | f | 70.8 | 20 | BostonSci | Genus RC | G | 7(30%), 8(70%) | 3.1 | 30 | 130 | G | 2(4%),3(4%), 4(32%),5(6%),6(6%), 7(48%) | 3.2 | 30 | 130 |  | |  |  |  |  |  | |  |  |  |  | 1125 | 480 |
| 129 | m | 66.0 | 12 | BostonSci | Genus R32 | G | 5(33%),6(34%),7(33%) | 2.4 | 60 | 185 | G | 1(25%), 5(24%),6(26%),7(25%) | 3.2 | 60 | 185 |  | |  |  |  |  |  | |  |  |  |  | 1400 | 520 |
| 130 | f | 63.9 | 21 | BostonSci | Genus RC | G | 1 (50%), 2-4(Rm 50%) | 3.8 | 60 | 130 | G | 3(30%),4(30%), 6(20%), 7(20%) | 2.7 | 60 | 130 |  | |  |  |  |  |  | |  |  |  |  | 1510 | 1250 |
| 131 | m | 52.6 | 5 | Medtronic | Percept | G | 2a,2b | 9.8 | 60 | 130 | G | 3 | 5 | 60 | 130 |  | |  |  |  |  |  | |  |  |  |  | 720 | 420 |
| 132 | m | 54.5 | 8 | BostonSci | Genus R32 | G | 1(35%), 2(35%), 5(15%) | 2.3 | 60 | 130 | G | 8(35%), 9(35%),11(15%), 12(15%) | 2.7 | 60 | 130 |  | |  |  |  |  |  | |  |  |  |  | 1340 | 100 |
| 133 | f | 68.8 | 13 | BostonSci | Genus R32 | G | 11(20%),12(60%),14(5%),15(15%) | 3 | 60 | 130 | G | 10(18%), 12(52%),13(8%), 15(22%) | 2.3 | 60 | 130 |  | |  |  |  |  |  | |  |  |  |  | 995 | 430 |
| 134 | m | 55.8 | 7 | BostonSci | Genus R32 | G | 10(28%),12(28%), 11(34%),13,15(3%each), 14(4%) | 3.8 | 60 | 185 | G | 7(14%),10(22%),12(64%), | 2.4 | 60 | 185 |  | |  |  |  |  |  | |  |  |  |  | 740 | 200 |
| 137 | f | 70.3 | 9 | BostonSci | Genus P16 | G | 2(25%),3(75%) | 2.7 | 60 | 130 | G | 2(75%),4(25%) | 2.5 | 60 | 130 |  | |  |  |  |  |  | |  |  |  |  | 480 | 100 |
| 139 | m | 64.3 | 15 | BostonSci | Genus P32 | G | 5(30%),8(70%) | 0.7 | 50 | 174 | G | 8(80%),11(20%) | 2.9 | 50 | 130 |  | |  |  |  |  |  | |  |  |  |  | 1750 | 550 |

Table S3: Per-patient subthalamic stimulation parameters at one-year and long-term (3–5-year) follow-up. Each patient is one row; contacts are given as configured clinically. Blank cells for the 3–5-year blocks indicate patients without a long-term follow-up visit.

| **Table S4: Attrition analysis: baseline and peri-operative characteristics of patients with versus without long-term (3–5-year) follow-up** | | | | |
| --- | --- | --- | --- | --- |
| **Characteristic** | **Completers (n = 33)** | **Non-completers (n = 67)** | **Test** | **p (FDR)** |
| Age (years) | 58.5 ± 7.1 | 61.1 ± 7.8 | Welch's t = −1.68 | 0.311 |
| Disease duration (years) | 11.0 [8.0–13.0] | 10.0 [8.0–13.0] | Mann–Whitney U = 1184.5 | 0.907 |
| MDS-UPDRS-III, pre-DBS | 46.0 [43.0–55.0] | 40.0 [35.0–47.0] | Mann–Whitney U = 1508 | 0.029 |
| MDS-UPDRS-III, post-DBS (1y-FU) | 20.0 [14.0–27.0] | 19.0 [15.0–25.5] | Mann–Whitney U = 1157 | 0.911 |
| MDS-UPDRS-II, pre-DBS | 12.5 [7.8–18.0] | 11.0 [8.0–15.0] | Mann–Whitney U = 1051.5 | 0.658 |
| MDS-UPDRS-II, post-DBS (1y-FU) | 8.0 [4.0–12.0] | 8.0 [4.5–14.0] | Mann–Whitney U = 851.5 | 0.907 |
| LEDD, pre-DBS (mg) | 1195.4 ± 567.4 | 1008.1 ± 449.9 | Welch's t = 1.66 | 0.311 |
| LEDD, post-DBS (1y-FU) (mg) | 467.5 [246.2–698.8] | 480.0 [300.0–660.0] | Mann–Whitney U = 1046.5 | 0.958 |
| Sex | F 11 (33%) / M 22 (67%) | F 21 (32%) / M 45 (68%) | χ² = 0.00 | 1.000 |

Table S4: Attrition analysis: baseline and peri-operative characteristics of patients with versus without long-term (3–5-year) follow-up. Completers (n = 33) are patients who completed the 3–5-year follow-up; non-completers (n = 67) are those who did not. Continuous variables were compared with Welch's t-test (reported as mean ± SD) or the Mann–Whitney U test (median [IQR]) according to distribution, and sex with the chi-square test; p-values were corrected across the nine characteristics using the Benjamini–Hochberg FDR procedure, and significance was judged on the adjusted value. p (FDR) = Benjamini–Hochberg-adjusted p across the nine characteristics. LEDD = levodopa-equivalent daily dose.

| **Table S5: Panel A. Cluster stability across candidate values of k** | | | |
| --- | --- | --- | --- |
| **k** | **Mean Jaccard** | **SD** | **Interpretation** |
| **2** | **0.77** | **0.12** | **Moderately stable** |
| 3 | 0.49 | 0.13 | Dissolved |
| 4 | 0.42 | 0.09 | Dissolved |
| 5 | 0.38 | 0.08 | Dissolved |
| 6 | 0.43 | 0.10 | Dissolved |
| 7 | 0.40 | 0.08 | Dissolved |
| 8 | 0.43 | 0.09 | Dissolved |
| 9 | 0.41 | 0.07 | Dissolved |
| 10 | 0.39 | 0.07 | Dissolved |

| **Table S5: Panel B. Per-cluster stability of the two-cluster solution** | | |
| --- | --- | --- |
| **Cluster (k = 2)** | **Mean Jaccard** | **SD** |
| Cluster 1 (higher impairment)† | 0.81 | 0.10 |
| Cluster 2 (lower impairment)† | 0.73 | 0.15 |

Table S5: Bootstrap assessment of cluster stability. Cluster reproducibility was quantified by non-parametric bootstrap resampling (B = 500) as the mean cluster-wise Jaccard similarity between the original partition and partitions obtained on resampled data (Hennig 2007). *Panel A* reports the overall mean Jaccard similarity for each candidate number of clusters (k = 2–10); the two-cluster solution was the only partition to avoid dissolution, corroborating the silhouette-based choice of k = 2. *Panel B* reports the per-cluster stability of the selected two-cluster solution. Interpretation follows Hennig's thresholds: stable ≥ 0.85; moderately stable 0.60–0.85; dissolved < 0.60.

| **Table S6: Descriptive between-profile comparison of the 22 baseline clustering features.** | | | | |
| --- | --- | --- | --- | --- |
| **Feature** | **Cluster 1 (n = 44) Mean (SD)** | **Cluster 2 (n = 56) Mean (SD)** | **Cohen's d** | **Magnitude** |
| Postural instability | 2.39 (1.06) | 0.57 (0.83) | **1.94** | **Large** |
| Gait | 2.73 (0.92) | 1.33 (0.72) | **1.72** | **Large** |
| Posture | 2.41 (0.82) | 1.06 (0.82) | **1.66** | **Large** |
| Mobility | 1.59 (1.30) | 0.41 (0.53) | **1.24** | **Large** |
| Bradykinesia | 23.89 (6.22) | 17.36 (5.59) | **1.11** | **Large** |
| Rigidity | 9.70 (3.37) | 6.59 (3.17) | **0.96** | **Large** |
| Anxiety | 0.90 (1.05) | 0.26 (0.61) | 0.78 | Medium |
| Freezing of gait | 1.61 (1.51) | 0.61 (1.09) | 0.77 | Medium |
| Psychomotor | 0.79 (1.13) | 0.30 (0.53) | 0.58 | Medium |
| Speech | 1.23 (0.86) | 0.79 (0.68) | 0.58 | Medium |
| Psychosomatic | 1.09 (1.20) | 0.55 (0.80) | 0.54 | Medium |
| Age (years) | 62.14 (7.51) | 58.14 (7.42) | 0.54 | Medium |
| Cognitive | 0.64 (1.06) | 0.22 (0.53) | 0.51 | Medium |
| Sex † | -0.09 (1.01) | -0.54 (0.85) | 0.48 | Small |
| Tremor | 4.32 (5.06) | 6.91 (6.23) | −0.45 | Small |
| Facial expression | 1.86 (0.73) | 1.59 (0.63) | 0.40 | Small |
| Affect | 0.73 (1.17) | 0.39 (0.75) | 0.35 | Small |
| Sleep | 1.93 (1.96) | 1.41 (1.61) | 0.29 | Small |
| Language | 0.66 (1.10) | 0.43 (0.67) | 0.26 | Small |
| Orientation | 0.15 (0.35) | 0.11 (0.27) | 0.15 | Negligible |
| Memory | 0.83 (1.17) | 0.92 (1.27) | −0.08 | Negligible |
| Attention | 0.12 (0.28) | 0.13 (0.38) | −0.03 | Negligible |

Table S6: Descriptive between-profile comparison of the 22 baseline clustering features: Descriptive between-profile comparison of the 22 baseline clustering features. Per-cluster means (SD) on the original scale and standardized mean differences (Cohen's d, Cluster 1 minus Cluster 2), ordered by absolute effect size. A positive d indicates higher scores in Cluster 1 (higher-impairment profile). Effect-size magnitude: |d| ≥ 0.8 large, ≥ 0.5 medium, ≥ 0.2 small, < 0.2 negligible. Separation is driven by axial and appendicular motor features (postural instability, gait, posture, mobility, bradykinesia, rigidity), consistent with PC1; tremor was higher in the lower-impairment profile (negative d).

† *Age and sex were included in the clustering feature set (age on its original scale; sex numerically encoded). Cluster 1 is the higher-impairment profile and Cluster 2 the lower-impairment profile. n = 100.*

| **Table S7: Distribution of the individual sub-domain scores at each timepoint.** | | | | | |
| --- | --- | --- | --- | --- | --- |
| **Feature** | **Timepoint** | **n** | **Mean** | **SD** | **Skewness** |
| mobility | Pre-DBS | 93 | 3.12 | 2.45 | 1.15 |
|  | 1-y FU | 92 | 1.82 | 1.85 | 1.07 |
|  | 3–5-y FU | 39 | 3.31 | 2.62 | 0.27 |
| gait | Pre-DBS | 96 | 4.50 | 2.98 | 0.55 |
|  | 1-y FU | 94 | 3.03 | 2.54 | 1.09 |
|  | 3–5-y FU | 39 | 3.95 | 2.84 | 0.55 |
| FoG | Pre-DBS | 96 | 2.26 | 2.10 | 0.67 |
|  | 1-y FU | 94 | 1.39 | 1.77 | 1.04 |
|  | 3–5-y FU | 39 | 2.31 | 1.75 | 0.28 |
| BAS | Pre-DBS | 99 | 2.86 | 2.30 | 1.21 |
|  | 1-y FU | 99 | 2.43 | 1.77 | 0.98 |
|  | 3–5-y FU | 39 | 2.10 | 1.47 | 0.82 |
| RAS | Pre-DBS | 100 | 1.30 | 1.16 | 1.26 |
|  | 1-y FU | 99 | 0.91 | 1.09 | 1.30 |
|  | 3–5-y FU | 39 | 0.69 | 1.00 | 1.66 |
| attention | Pre-DBS | 100 | 5.87 | 0.34 | −3.23 |
|  | 1-y FU | 100 | 5.76 | 0.62 | −3.85 |
|  | 3–5-y FU | 39 | 5.65 | 0.95 | −3.83 |
| language | Pre-DBS | 100 | 5.47 | 0.89 | −3.05 |
|  | 1-y FU | 100 | 5.34 | 0.78 | −0.99 |
|  | 3–5-y FU | 39 | 5.12 | 0.87 | −0.75 |
| memory | Pre-DBS | 98 | 4.12 | 1.23 | −1.62 |
|  | 1-y FU | 100 | 4.17 | 1.11 | −1.64 |
|  | 3–5-y FU | 39 | 3.39 | 1.65 | −0.71 |
| orientation | Pre-DBS | 98 | 5.87 | 0.31 | −2.32 |
|  | 1-y FU | 100 | 5.85 | 0.32 | −2.15 |
|  | 3–5-y FU | 39 | 5.62 | 0.98 | −3.51 |
| affect | Pre-DBS | 98 | 0.54 | 0.98 | 2.67 |
|  | 1-y FU | 98 | 0.46 | 0.84 | 2.16 |
|  | 3–5-y FU | 40 | 1.50 | 1.60 | 0.57 |
| anxiety | Pre-DBS | 98 | 0.75 | 1.02 | 1.31 |
|  | 1-y FU | 98 | 0.51 | 0.86 | 1.73 |
|  | 3–5-y FU | 40 | 0.83 | 1.13 | 1.37 |
| psychosomatic | Pre-DBS | 97 | 0.33 | 0.55 | 1.46 |
|  | 1-y FU | 97 | 0.29 | 0.50 | 1.45 |
|  | 3–5-y FU | 40 | 0.70 | 0.82 | 0.62 |
| psychomotor | Pre-DBS | 98 | 0.51 | 0.89 | 2.13 |
|  | 1-y FU | 97 | 0.67 | 0.98 | 2.08 |
|  | 3–5-y FU | 40 | 0.88 | 0.76 | 0.96 |

Table S7: Distribution of the individual sub-domain scores at each timepoint (n, mean, SD, skewness). Positive skew indicates a floor effect; negative skew a ceiling effect.

| **Table S8: Prediction models of improvement induced by STN-DBS from baseline scores for each feature:** | | | | | | |
| --- | --- | --- | --- | --- | --- | --- |
| **Feature** | **R²** | **Coef.** | **t-value** | **p-value** | **FDR-p** | **p (FDR)** |
| **Mobility** | 0.489 | 0.666 | 9.702 | 5.359623155015897e-16 | 1.7418775253801665e-15 | **TRUE** |
| **Gait** | 0.373 | 0.560 | 7.645 | 1.4392925531270802e-11 | 2.0789781322946714e-11 | **TRUE** |
| **FoG** | 0.367 | 0.535 | 7.547 | 2.3120039363602816e-11 | 3.005605117268366e-11 | **TRUE** |
| **BAS** | 0.606 | 0.957 | 12.301 | 1.3784923004289289e-21 | 1.7920399905576074e-20 | **TRUE** |
| **RAS** | 0.542 | 1.017 | 10.776 | 2.5189771380091838e-18 | 1.0915567598039795e-17 | **TRUE** |
| **Attention** | 0.098 | 0.588 | 3.272 | 0.001475006105054166 | 0.001475006105054166 | **TRUE** |
| **Language** | 0.378 | 0.613 | 7.727 | 9.608420321034575e-12 | 1.5613683021681184e-11 | **TRUE** |
| **Memery** | 0.428 | 0.749 | 8.565 | 1.549864752518648e-13 | 2.8783202546774893e-13 | **TRUE** |
| **Orientation** | 0.287 | 0.625 | 6.293 | 8.74941199982374e-09 | 9.478529666475717e-09 | **TRUE** |
| **Affect** | 0.459 | 0.761 | 9.121 | 9.736669869038907e-15 | 2.5315341659501155e-14 | **TRUE** |
| **Annxiety** | 0.574 | 0.985 | 11.514 | 6.510587752081463e-20 | 4.2318820388529507e-19 | **TRUE** |
| **Psychosomatic** | 0.451 | 0.797 | 8.988 | 1.897773551380831e-14 | 4.111842694658467e-14 | **TRUE** |
| **Psychomotor** | 0.341 | 0.772 | 7.124 | 1.7761451216654867e-10 | 2.0990805983319388e-10 | **TRUE** |

Table S8: Prediction models of STN-DBS-induced improvement from baseline scores for each feature. Linear regression of DBS-induced change on baseline severity for each sub-domain, reporting R², coefficient, t-value, uncorrected p-value, FDR-corrected p-value and significance. FoG: Freezing of gait, BAS: Bradykinesia assymetry score, RAS: Ridgidity assymetry score.

| **Table S9:** Complete moderation analysis (68 models). | | | | | | | | | | | |
| --- | --- | --- | --- | --- | --- | --- | --- | --- | --- | --- | --- |
| **Predictor** | **Moderator** | **R²** | **Model p** | **Predictor coef** | **Predictor p** | **Moderator coef** | **Moderator p** | **Interaction coef** | **Interaction 95% CI** | **Interaction p** | **Interaction p (FDR)** |
| **ANCOVA: baseline moderators (_1)** | | | | | | | | | | | |
| mobility_1 | attention_1 | 0.263 | <0.001 | 0.264 | <0.001 | 0.276 | 0.246 | 1.567 | [−0.080, 3.214] | 0.062 | 0.124 |
| **mobility_1** | **language_1** | **0.298** | **<0.001** | **0.303** | **<0.001** | **0.160** | **0.081** | **1.724** | **[0.535, 2.913]** | **0.005** | **0.020** |
| mobility_1 | memory_1 | 0.252 | <0.001 | 0.298 | <0.001 | −0.077 | 0.173 | −0.352 | [−0.941, 0.238] | 0.239 | 0.340 |
| mobility_1 | orientation_1 | 0.238 | <0.001 | 0.293 | <0.001 | −0.087 | 0.745 | −2.167 | [−5.925, 1.592] | 0.255 | 0.340 |
| mobility_1 | affect_1 | 0.232 | <0.001 | 0.278 | <0.001 | 0.050 | 0.687 | 0.180 | [−0.629, 0.989] | 0.660 | 0.660 |
| mobility_1 | anxiety_1 | 0.233 | <0.001 | 0.289 | <0.001 | −0.005 | 0.963 | 0.430 | [−0.596, 1.455] | 0.408 | 0.466 |
| **mobility_1** | **psychosomatic_1** | **0.294** | **<0.001** | **0.317** | **<0.001** | **0.080** | **0.415** | **1.309** | **[0.307, 2.311]** | **0.011** | **0.029** |
| **mobility_1** | **psychomotor_1** | **0.32** | **<0.001** | **0.342** | **<0.001** | **0.094** | **0.289** | **2.159** | **[0.848, 3.470]** | **0.002** | **0.012** |
| gait_1 | attention_1 | 0.293 | <0.001 | 0.381 | <0.001 | 0.018 | 0.955 | −0.356 | [−3.580, 2.868] | 0.827 | 0.941 |
| gait_1 | language_1 | 0.298 | <0.001 | 0.391 | <0.001 | 0.039 | 0.749 | 0.513 | [−0.734, 1.760] | 0.416 | 0.941 |
| gait_1 | memory_1 | 0.292 | <0.001 | 0.384 | <0.001 | 0.003 | 0.972 | 0.023 | [−0.582, 0.627] | 0.941 | 0.941 |
| gait_1 | orientation_1 | 0.293 | <0.001 | 0.385 | <0.001 | −0.023 | 0.948 | −0.242 | [−3.163, 2.678] | 0.870 | 0.941 |
| gait_1 | affect_1 | 0.3 | <0.001 | 0.385 | <0.001 | −0.147 | 0.332 | 0.072 | [−0.964, 1.107] | 0.891 | 0.941 |
| gait_1 | anxiety_1 | 0.293 | <0.001 | 0.384 | <0.001 | 0.025 | 0.864 | 0.072 | [−1.182, 1.327] | 0.909 | 0.941 |
| gait_1 | psychosomatic_1 | 0.311 | <0.001 | 0.413 | <0.001 | 0.004 | 0.978 | 1.021 | [−0.257, 2.300] | 0.116 | 0.928 |
| gait_1 | psychomotor_1 | 0.295 | <0.001 | 0.390 | <0.001 | −0.067 | 0.653 | 0.258 | [−0.763, 1.279] | 0.617 | 0.941 |
| fog_1 | attention_1 | 0.305 | <0.001 | 0.466 | <0.001 | −0.037 | 0.913 | 0.238 | [−2.127, 2.604] | 0.842 | 0.988 |
| fog_1 | language_1 | 0.315 | <0.001 | 0.460 | <0.001 | −0.126 | 0.350 | 0.620 | [−0.557, 1.797] | 0.298 | 0.988 |
| fog_1 | memory_1 | 0.309 | <0.001 | 0.469 | <0.001 | −0.047 | 0.565 | 0.074 | [−0.521, 0.669] | 0.805 | 0.988 |
| fog_1 | orientation_1 | 0.32 | <0.001 | 0.478 | <0.001 | 0.176 | 0.634 | −1.756 | [−4.448, 0.937] | 0.199 | 0.988 |
| fog_1 | affect_1 | 0.311 | <0.001 | 0.461 | <0.001 | 0.004 | 0.981 | −0.655 | [−2.111, 0.802] | 0.374 | 0.988 |
| fog_1 | anxiety_1 | 0.308 | <0.001 | 0.465 | <0.001 | 0.086 | 0.568 | −0.122 | [−1.533, 1.289] | 0.864 | 0.988 |
| fog_1 | psychosomatic_1 | 0.308 | <0.001 | 0.479 | <0.001 | 0.088 | 0.523 | 0.169 | [−1.357, 1.695] | 0.827 | 0.988 |
| fog_1 | psychomotor_1 | 0.334 | <0.001 | 0.441 | <0.001 | 0.258 | 0.081 | −0.004 | [−0.820, 0.811] | 0.992 | 0.992 |
| motor_1 | attention_1 | 0.319 | <0.001 | 0.422 | <0.001 | 0.071 | 0.773 | 0.702 | [−1.417, 2.821] | 0.512 | 0.819 |
| motor_1 | language_1 | 0.332 | <0.001 | 0.428 | <0.001 | 0.017 | 0.852 | 0.967 | [−0.320, 2.253] | 0.139 | 0.556 |
| motor_1 | memory_1 | 0.32 | <0.001 | 0.426 | <0.001 | −0.038 | 0.526 | 0.056 | [−0.496, 0.607] | 0.841 | 0.961 |
| motor_1 | orientation_1 | 0.324 | <0.001 | 0.430 | <0.001 | 0.002 | 0.994 | −1.611 | [−4.717, 1.494] | 0.306 | 0.611 |
| motor_1 | affect_1 | 0.317 | <0.001 | 0.428 | <0.001 | −0.039 | 0.743 | −0.007 | [−0.964, 0.950] | 0.989 | 0.989 |
| motor_1 | anxiety_1 | 0.317 | <0.001 | 0.426 | <0.001 | 0.030 | 0.788 | 0.131 | [−1.059, 1.321] | 0.827 | 0.961 |
| motor_1 | psychosomatic_1 | 0.348 | <0.001 | 0.480 | <0.001 | 0.055 | 0.578 | 1.278 | [0.020, 2.536] | 0.046 | 0.372 |
| motor_1 | psychomotor_1 | 0.335 | <0.001 | 0.412 | <0.001 | 0.062 | 0.572 | 0.494 | [−0.445, 1.432] | 0.299 | 0.611 |
| **ANCOVA: post-DBS moderators (_2)** | | | | | | | | | | | |
| mobility_1 | attention_2 | 0.247 | <0.001 | 0.283 | <0.001 | −0.148 | 0.366 | 0.953 | [−0.225, 2.130] | 0.112 | 0.179 |
| mobility_1 | language_2 | 0.256 | <0.001 | 0.305 | <0.001 | −0.099 | 0.340 | −0.920 | [−2.036, 0.195] | 0.105 | 0.179 |
| mobility_1 | memory_2 | 0.255 | <0.001 | 0.336 | <0.001 | −0.081 | 0.277 | −0.217 | [−0.664, 0.231] | 0.339 | 0.387 |
| mobility_1 | orientation_2 | 0.253 | <0.001 | 0.280 | <0.001 | −0.324 | 0.227 | 1.509 | [−0.291, 3.308] | 0.099 | 0.179 |
| mobility_1 | affect_2 | 0.294 | <0.001 | 0.192 | 0.022 | 0.310 | 0.035 | 0.468 | [−0.334, 1.270] | 0.250 | 0.333 |
| **mobility_1** | **anxiety_2** | **0.373** | **<0.001** | **0.268** | **<0.001** | **0.179** | **0.137** | **2.805** | **[1.378, 4.233]** | **<0.001** | **0.001** |
| **mobility_1** | **psychosomatic_2** | **0.326** | **<0.001** | **0.228** | **0.002** | **0.118** | **0.275** | **1.491** | **[0.574, 2.409]** | **0.002** | **0.007** |
| mobility_1 | psychomotor_2 | 0.234 | <0.001 | 0.279 | <0.001 | 0.083 | 0.345 | −0.015 | [−0.720, 0.690] | 0.966 | 0.966 |
| gait_1 | attention_2 | 0.308 | <0.001 | 0.364 | <0.001 | 0.130 | 0.568 | −1.349 | [−3.278, 0.581] | 0.168 | 0.674 |
| gait_1 | language_2 | 0.295 | <0.001 | 0.386 | <0.001 | 0.088 | 0.529 | −0.098 | [−1.220, 1.025] | 0.863 | 0.970 |
| gait_1 | memory_2 | 0.305 | <0.001 | 0.380 | <0.001 | 0.109 | 0.257 | −0.291 | [−0.908, 0.327] | 0.352 | 0.727 |
| gait_1 | orientation_2 | 0.293 | <0.001 | 0.380 | <0.001 | 0.080 | 0.829 | 0.050 | [−2.533, 2.632] | 0.970 | 0.970 |
| gait_1 | affect_2 | 0.294 | <0.001 | 0.383 | <0.001 | 0.078 | 0.662 | −0.045 | [−1.234, 1.143] | 0.940 | 0.970 |
| gait_1 | anxiety_2 | 0.377 | <0.001 | 0.424 | <0.001 | 0.318 | 0.051 | 2.055 | [0.527, 3.583] | 0.009 | 0.071 |
| gait_1 | psychosomatic_2 | 0.306 | <0.001 | 0.390 | <0.001 | 0.156 | 0.290 | 0.479 | [−0.657, 1.615] | 0.405 | 0.727 |
| gait_1 | psychomotor_2 | 0.309 | <0.001 | 0.366 | <0.001 | 0.124 | 0.285 | 0.338 | [−0.555, 1.231] | 0.454 | 0.727 |
| fog_1 | attention_2 | 0.306 | <0.001 | 0.471 | <0.001 | 0.074 | 0.731 | 0.109 | [−2.756, 2.973] | 0.940 | 0.961 |
| fog_1 | language_2 | 0.306 | <0.001 | 0.466 | <0.001 | −0.049 | 0.735 | −0.028 | [−1.138, 1.083] | 0.961 | 0.961 |
| fog_1 | memory_2 | 0.37 | <0.001 | 0.511 | <0.001 | −0.188 | 0.050 | −0.650 | [−1.357, 0.057] | 0.071 | 0.570 |
| fog_1 | orientation_2 | 0.319 | <0.001 | 0.442 | <0.001 | 0.573 | 0.176 | −0.591 | [−3.128, 1.945] | 0.644 | 0.961 |
| fog_1 | affect_2 | 0.321 | <0.001 | 0.464 | <0.001 | 0.179 | 0.333 | 0.656 | [−0.630, 1.942] | 0.314 | 0.858 |
| fog_1 | anxiety_2 | 0.313 | <0.001 | 0.454 | <0.001 | 0.016 | 0.938 | 0.547 | [−0.690, 1.783] | 0.382 | 0.858 |
| fog_1 | psychosomatic_2 | 0.307 | <0.001 | 0.467 | <0.001 | −0.087 | 0.576 | 0.140 | [−1.074, 1.354] | 0.819 | 0.961 |
| fog_1 | psychomotor_2 | 0.338 | <0.001 | 0.426 | <0.001 | 0.216 | 0.066 | 0.335 | [−0.502, 1.172] | 0.429 | 0.858 |
| motor_1 | attention_2 | 0.317 | <0.001 | 0.433 | <0.001 | −0.053 | 0.770 | 0.433 | [−1.448, 2.314] | 0.649 | 0.649 |
| motor_1 | language_2 | 0.322 | <0.001 | 0.436 | <0.001 | −0.018 | 0.869 | −0.464 | [−1.536, 0.608] | 0.392 | 0.617 |
| motor_1 | memory_2 | 0.339 | <0.001 | 0.452 | <0.001 | −0.053 | 0.479 | −0.360 | [−0.918, 0.198] | 0.203 | 0.541 |
| motor_1 | orientation_2 | 0.321 | <0.001 | 0.412 | <0.001 | −0.009 | 0.977 | 0.838 | [−1.443, 3.120] | 0.467 | 0.617 |
| motor_1 | affect_2 | 0.332 | <0.001 | 0.401 | <0.001 | 0.161 | 0.257 | 0.302 | [−0.672, 1.276] | 0.540 | 0.617 |
| **motor_1** | **anxiety_2** | **0.419** | **<0.001** | **0.418** | **<0.001** | **0.074** | **0.577** | **2.298** | **[0.974, 3.623]** | **<0.001** | **0.007** |
| motor_1 | psychosomatic_2 | 0.354 | <0.001 | 0.401 | <0.001 | 0.051 | 0.650 | 1.186 | [0.134, 2.237] | 0.028 | 0.110 |
| motor_1 | psychomotor_2 | 0.342 | <0.001 | 0.385 | <0.001 | 0.124 | 0.162 | 0.366 | [−0.374, 1.107] | 0.329 | 0.617 |
| **Composite domains: baseline (_1)** | | | | | | | | | | | |
| motor_1 | cognition_1 | 0.322 | <0.001 | 0.426 | <0.001 | −0.059 | 0.762 | 0.874 | [−1.807, 3.554] | 0.523 | 0.523 |
| motor_1 | mood_1 | 0.338 | <0.001 | 0.422 | <0.001 | 0.097 | 0.473 | 1.450 | [−0.222, 3.121] | 0.089 | 0.178 |
| **Composite domains: post-DBS (_2)** | | | | | | | | | | | |
| motor_1 | cognition_2 | 0.327 | <0.001 | 0.442 | <0.001 | −0.068 | 0.716 | −0.729 | [−3.292, 1.833] | 0.577 | 0.577 |
| **motor_1** | **mood_2** | **0.39** | **<0.001** | **0.354** | **<0.001** | **0.248** | **0.151** | **1.725** | **[0.553, 2.896]** | **0.004** | **0.008** |

Table S9: Complete moderation analysis (68 models). Each ANCOVA model predicted the one-year value of a motor outcome from its pre-DBS baseline value (predictor), a non-motor moderator, and their interaction (predictor × moderator). Models are grouped by analysis family. Interaction p-values were corrected for multiple comparisons using the Benjamini–Hochberg FDR procedure as described in Methods; rows with a statistically significant interaction after correction are shown in bold. Suffixes denote timepoint (_1 = pre-DBS baseline; _2 = one-year follow-up).

| **Table S10:** Regression diagnostics for the 68 moderation models. | | | | | | | | | | |
| --- | --- | --- | --- | --- | --- | --- | --- | --- | --- | --- |
| **Predictor** | **Moderator** | **Outcome** | **n** | **RESET F** | **RESET p** | **BP LM** | **BP p** | **Max Cook's D** | **N influential** | **Max VIF** |
| **ANCOVA, baseline moderators (_1)** | | | | | | | | | | |
| mobility_1 | attention_1 | mobility_2 | 100 | 0.439 | 0.646 | 22.314 | <0.001 | 0.315 | 8 | 1.984 |
| mobility_1 | language_1 | mobility_2 | 100 | 0.196 | 0.822 | 17.154 | 0.004 | 0.241 | 6 | 4.108 |
| mobility_1 | memory_1 | mobility_2 | 100 | 1.143 | 0.323 | 22.096 | 0.001 | 1.574 | 10 | 3.546 |
| mobility_1 | orientation_1 | mobility_2 | 100 | 0.101 | 0.904 | 30.247 | <0.001 | 0.341 | 10 | 4.771 |
| mobility_1 | affect_1 | mobility_2 | 100 | 0.378 | 0.686 | 28.985 | <0.001 | 1.669 | 8 | 4.000 |
| mobility_1 | anxiety_1 | mobility_2 | 100 | 0.708 | 0.495 | 26.379 | <0.001 | 0.423 | 9 | 4.301 |
| mobility_1 | psychosomatic_1 | mobility_2 | 100 | 0.256 | 0.775 | 23.366 | <0.001 | 0.342 | 9 | 3.995 |
| mobility_1 | psychomotor_1 | mobility_2 | 100 | 0.202 | 0.817 | 13.028 | 0.023 | 0.567 | 8 | 6.094 |
| gait_1 | attention_1 | gait_2 | 100 | 3.435 | 0.036 | 6.213 | 0.286 | 0.272 | 9 | 5.180 |
| gait_1 | language_1 | gait_2 | 100 | 2.699 | 0.073 | 6.863 | 0.231 | 0.339 | 9 | 4.756 |
| gait_1 | memory_1 | gait_2 | 100 | 3.304 | 0.041 | 6.969 | 0.223 | 0.106 | 9 | 3.106 |
| gait_1 | orientation_1 | gait_2 | 100 | 3.625 | 0.031 | 6.953 | 0.224 | 0.351 | 10 | 3.399 |
| gait_1 | affect_1 | gait_2 | 100 | 3.444 | 0.036 | 7.845 | 0.165 | 0.192 | 9 | 3.913 |
| gait_1 | anxiety_1 | gait_2 | 100 | 4.462 | 0.014 | 7.605 | 0.179 | 0.658 | 7 | 4.950 |
| gait_1 | psychosomatic_1 | gait_2 | 100 | 2.265 | 0.110 | 6.462 | 0.264 | 0.161 | 9 | 4.588 |
| gait_1 | psychomotor_1 | gait_2 | 100 | 3.355 | 0.039 | 7.095 | 0.214 | 0.181 | 9 | 6.697 |
| fog_1 | attention_1 | fog_2 | 100 | 2.082 | 0.131 | 18.784 | 0.002 | 0.401 | 5 | 1.833 |
| fog_1 | language_1 | fog_2 | 100 | 0.664 | 0.517 | 24.142 | <0.001 | 0.340 | 7 | 4.803 |
| fog_1 | memory_1 | fog_2 | 100 | 1.850 | 0.163 | 19.257 | 0.002 | 0.134 | 8 | 2.245 |
| fog_1 | orientation_1 | fog_2 | 100 | 1.938 | 0.150 | 19.103 | 0.002 | 0.130 | 7 | 2.106 |
| fog_1 | affect_1 | fog_2 | 100 | 1.258 | 0.289 | 17.638 | 0.003 | 0.464 | 8 | 3.732 |
| fog_1 | anxiety_1 | fog_2 | 100 | 1.473 | 0.235 | 18.436 | 0.002 | 0.086 | 9 | 3.446 |
| fog_1 | psychosomatic_1 | fog_2 | 100 | 1.164 | 0.317 | 19.369 | 0.002 | 0.198 | 9 | 3.555 |
| fog_1 | psychomotor_1 | fog_2 | 100 | 0.586 | 0.559 | 16.311 | 0.006 | 0.884 | 7 | 3.972 |
| motor_1 | attention_1 | motor_2 | 100 | 0.782 | 0.461 | 14.287 | 0.014 | 0.733 | 9 | 2.748 |
| motor_1 | language_1 | motor_2 | 100 | 0.518 | 0.598 | 11.524 | 0.042 | 0.104 | 8 | 6.592 |
| motor_1 | memory_1 | motor_2 | 100 | 0.674 | 0.512 | 14.667 | 0.012 | 0.624 | 9 | 3.125 |
| motor_1 | orientation_1 | motor_2 | 100 | 0.883 | 0.417 | 16.137 | 0.006 | 0.111 | 7 | 4.012 |
| motor_1 | affect_1 | motor_2 | 100 | 0.496 | 0.611 | 14.627 | 0.012 | 0.941 | 7 | 4.289 |
| motor_1 | anxiety_1 | motor_2 | 100 | 0.681 | 0.509 | 14.336 | 0.014 | 0.451 | 5 | 5.095 |
| motor_1 | psychosomatic_1 | motor_2 | 100 | 0.183 | 0.833 | 13.532 | 0.019 | 0.130 | 7 | 5.375 |
| motor_1 | psychomotor_1 | motor_2 | 100 | 0.813 | 0.447 | 11.316 | 0.045 | 0.271 | 8 | 6.429 |
| **ANCOVA, post-DBS moderators (_2)** | | | | | | | | | | |
| mobility_1 | attention_2 | mobility_2 | 100 | 0.143 | 0.867 | 28.798 | <0.001 | 0.240 | 7 | 5.294 |
| mobility_1 | language_2 | mobility_2 | 100 | 0.479 | 0.621 | 32.652 | <0.001 | 0.947 | 9 | 4.236 |
| mobility_1 | memory_2 | mobility_2 | 100 | 0.275 | 0.760 | 29.798 | <0.001 | 2.396 | 10 | 4.397 |
| mobility_1 | orientation_2 | mobility_2 | 100 | 2.289 | 0.107 | 26.081 | <0.001 | 6.823 | 7 | 3.234 |
| mobility_1 | affect_2 | mobility_2 | 100 | 0.987 | 0.377 | 17.505 | 0.004 | 0.225 | 9 | 3.955 |
| mobility_1 | anxiety_2 | mobility_2 | 100 | 0.414 | 0.662 | 14.688 | 0.012 | 0.193 | 10 | 5.576 |
| mobility_1 | somatic_2 | mobility_2 | 100 | 1.067 | 0.348 | 11.252 | 0.047 | 0.207 | 7 | 3.966 |
| mobility_1 | psychomotor_2 | mobility_2 | 100 | 0.268 | 0.766 | 32.038 | <0.001 | 0.542 | 11 | 3.763 |
| gait_1 | attention_2 | gait_2 | 100 | 2.844 | 0.063 | 5.174 | 0.395 | 0.895 | 10 | 10.152 |
| gait_1 | language_2 | gait_2 | 100 | 3.365 | 0.039 | 6.598 | 0.252 | 0.115 | 9 | 4.342 |
| gait_1 | memory_2 | gait_2 | 100 | 3.458 | 0.036 | 6.784 | 0.237 | 0.105 | 9 | 5.294 |
| gait_1 | orientation_2 | gait_2 | 100 | 3.142 | 0.048 | 8.064 | 0.153 | 0.115 | 10 | 5.234 |
| gait_1 | affect_2 | gait_2 | 100 | 3.184 | 0.046 | 6.908 | 0.228 | 0.127 | 9 | 3.424 |
| gait_1 | anxiety_2 | gait_2 | 100 | 0.991 | 0.375 | 2.728 | 0.742 | 0.300 | 8 | 5.120 |
| gait_1 | psychosomatic_2 | gait_2 | 100 | 3.371 | 0.039 | 5.276 | 0.383 | 0.292 | 7 | 3.553 |
| gait_1 | psychomotor_2 | gait_2 | 100 | 1.604 | 0.207 | 5.827 | 0.323 | 0.107 | 8 | 4.964 |
| fog_1 | attention_2 | fog_2 | 100 | 2.215 | 0.115 | 18.097 | 0.003 | 0.145 | 6 | 9.411 |
| fog_1 | language_2 | fog_2 | 100 | 2.362 | 0.100 | 19.270 | 0.002 | 0.124 | 8 | 2.979 |
| fog_1 | memory_2 | fog_2 | 100 | 1.801 | 0.171 | 15.026 | 0.010 | 0.112 | 8 | 4.841 |
| fog_1 | orientation_2 | fog_2 | 100 | 1.878 | 0.159 | 23.480 | <0.001 | 0.296 | 7 | 4.477 |
| fog_1 | affect_2 | fog_2 | 100 | 1.409 | 0.250 | 15.222 | 0.009 | 0.082 | 6 | 2.657 |
| fog_1 | anxiety_2 | fog_2 | 100 | 1.560 | 0.216 | 20.206 | 0.001 | 0.085 | 8 | 4.359 |
| fog_1 | psychosomatic_2 | fog_2 | 100 | 1.750 | 0.180 | 21.345 | 0.001 | 0.133 | 10 | 3.564 |
| fog_1 | psychomotor_2 | fog_2 | 100 | 1.258 | 0.289 | 15.749 | 0.008 | 0.222 | 5 | 3.603 |
| motor_1 | attention_2 | motor_2 | 100 | 0.343 | 0.710 | 13.185 | 0.022 | 0.344 | 8 | 11.087 |
| motor_1 | language_2 | motor_2 | 100 | 0.633 | 0.534 | 15.780 | 0.008 | 0.279 | 9 | 4.460 |
| motor_1 | memory_2 | motor_2 | 100 | 1.603 | 0.207 | 14.239 | 0.014 | 0.666 | 6 | 5.733 |
| motor_1 | orientation_2 | motor_2 | 100 | 0.786 | 0.459 | 15.541 | 0.008 | 0.399 | 8 | 5.511 |
| motor_1 | affect_2 | motor_2 | 100 | 1.006 | 0.370 | 11.863 | 0.037 | 0.282 | 7 | 3.634 |
| motor_1 | anxiety_2 | motor_2 | 100 | 1.353 | 0.263 | 6.155 | 0.291 | 0.466 | 5 | 6.442 |
| motor_1 | psychosomatic_2 | motor_2 | 100 | 0.301 | 0.741 | 8.520 | 0.130 | 0.197 | 6 | 4.479 |
| motor_1 | psychomotor_2 | motor_2 | 100 | 1.125 | 0.329 | 12.588 | 0.028 | 0.190 | 9 | 4.525 |
| **Composite domains, baseline moderators (_1)** | | | | | | | | | | |
| motor_1 | cognition_1 | motor_2 | 100 | 0.711 | 0.494 | 13.730 | 0.017 | 0.259 | 7 | 4.676 |
| motor_1 | mood_1 | motor_2 | 100 | 0.858 | 0.427 | 11.909 | 0.036 | 0.096 | 7 | 6.900 |
| **Composite domains, post-DBS moderators (_2)** | | | | | | | | | | |
| motor_1 | cognition_2 | motor_2 | 100 | 1.384 | 0.256 | 15.726 | 0.015 | 0.405 | 7 | 6.619 |
| motor_1 | mood_2 | motor_2 | 100 | 0.416 | 0.661 | 6.884 | 0.332 | 0.111 | 5 | 5.973 |

Table S10: Regression diagnostics for the 68 moderation models (companion to table S9). For each model we report the Ramsey RESET test for functional-form misspecification (F, p), the Breusch–Pagan test for heteroscedasticity (LM statistic, p), the maximum Cook's distance and the number of influential observations, and the maximum variance inflation factor (VIF). Heteroscedasticity was addressed by using HC3 heteroscedasticity-consistent standard errors, and multicollinearity among the interaction constituents was minimised by mean-centring the predictor and moderator before forming the interaction term (maximum VIF 11.1; nearly all models below 10). RESET flags were confined to a subset of gait models and did not change the significance of the reported interactions. RESET = Ramsey Regression Equation Specification Error Test (functional form); BP = Breusch–Pagan test (heteroscedasticity), reported as the LM statistic and its p-value; Max Cook's D = largest Cook's distance across observations; N influential = number of observations exceeding the Cook's distance threshold (as defined in Methods); Max VIF = largest variance inflation factor among model terms. p-values are reported as <0.001 where below that threshold. Predictor and moderator suffixes denote timepoint (_1 = pre-DBS baseline, _2 = one-year follow-up); Outcome is the one-year value of the motor feature.

# **Supplementary Figures:**

**Fig. S1: Patient's exclusion criteria.**

**Fig. S2 Principal component analysis (PCA) structure underlying the baseline clustering.** (A) Loadings of the 22 baseline clinical sub-domain features on the first 12 principal components (PCs), which together explain 82.9% of the variance; only loadings with |loading| ≥ 0.20 are shown, and features are grouped by domain (motor, cognitive, mood). PC1 (19.8% of variance) is dominated by axial and appendicular motor features — gait (0.39), mobility (0.36), postural instability (0.35), posture (0.34), bradykinesia (0.29), and rigidity (0.27) — identifying it as a global motor-severity axis; higher-order components capture more specific cognitive (e.g., memory, PC6 = 0.61) and mood (e.g., psychomotor, PC4 = 0.59) contributions. (B) Cumulative variance explained as a function of the number of components; the horizontal line marks the 99.9% threshold applied before k-means clustering, corresponding to the 22 components. (C) Radar plot of the mean PC scores (PC1–PC22) for the two clusters at pre-DBS baseline. The clusters separate almost entirely along PC1 (Cluster 1 ≫ Cluster 2), with substantial overlap on all remaining components, indicating that the partition primarily reflects a single continuous dimension of overall motor severity rather than a distinct multivariate phenotype. Cluster 1 = higher-impairment profile (n = 44); Cluster 2 = lower-impairment profile (n = 56). FoG = freezing of gait; PC = principal component.

**Fig. S3 Distributions of the individual sub-domain scores by timepoint.** Violin plots with superimposed box plots and individual data points for each sub-domain score at pre-DBS, 1-year, and 3–5-year follow-up. Motor and mood sub-scores were right-skewed with floor effects, whereas cognitive sub-scores were left-skewed with ceiling effects (Table S6). Brackets show per-feature longitudinal comparisons; for each comparison the normality of the paired differences was assessed (Shapiro–Wilk) and a Wilcoxon signed-rank test used where they departed from normality (paired t-test otherwise), with Benjamini–Hochberg FDR correction across the 39 comparisons. *** FDR p < .001; ** FDR p < .01; * FDR p < .05; n.s. = not significant. BAS = bradykinesia asymmetry score; RAS = rigidity asymmetry score; FoG = freezing of gait.

**Fig. S4** **Linear mixed-effects timepoint contrasts for the first three principal components: full cohort versus pre-existing MCI subgroup.** Each row shows the estimated change from pre-DBS baseline (β) for one principal component and timepoint contrast; points are point estimates and whiskers are 95% confidence intervals. Blue = full cohort (n = 100); orange = MCI subgroup (n = 16; pre-DBS MoCA < 26 or Mattis DRS < 136). The dashed vertical line at β = 0 denotes no change from baseline; estimates to the left of the line indicate a lower component score at one-year follow-up. PC scores were projected onto the full-cohort PCA axes and the identical mixed model was re-fitted within the subgroup, so the two sets of coefficients are on the same scale and directly comparable. Only the pre-DBS-to-one-year contrast is displayed, as the subgroup contained no long-term (3–5-year) follow-up visits; the corresponding 3–5-year rows are shown for completeness but left blank. PC1 = motor-severity axis; PC2 = memory. The one-year reduction in PC1 reached significance in both the full cohort and the MCI subgroup, indicating that motor improvement was preserved in cognitively impaired patients, whereas changes in PC2 and PC3 did not reach significance within the subgroup. Because the subgroup comprised 16 patients, these analyses are exploratory and underpowered, and the overlapping confidence intervals were not formally tested for a group × time interaction.

**Fig. S5 Medication (ΔLEDD) sensitivity analysis.** (A) Spearman rank correlations between the change in levodopa-equivalent daily dose (ΔLEDD, pre-DBS to one-year follow-up) and the corresponding change in each clinical sub-domain score. No feature showed a statistically significant association with ΔLEDD after Benjamini–Hochberg FDR correction, providing no evidence that the clinical change scores were systematically related to the magnitude of medication reduction. (B) Difference in principal-component loadings between the original one-year follow-up PCA and a PCA computed after residualising each feature on ΔLEDD (Procrustes distance = 0.004); only loadings whose absolute change exceeded 0.05 are displayed. No loading exceeded this threshold on PC1–PC3, indicating that the component structure was essentially unchanged after removing linear ΔLEDD-related variance. FoG = freezing of gait; LEDD = levodopa-equivalent daily dose; PC = principal component.

1.	Tomlinson CL, Stowe R, Patel S, et al (2010) Systematic review of levodopa dose equivalency reporting in Parkinson’s disease. Mov Disord 25:2649–2653. https://doi.org/10.1002/mds.23429

2.	Goetz CG, Tilley BC, Shaftman SR, et al (2008) Movement Disorder Society-sponsored revision of the Unified Parkinson’s Disease Rating Scale (MDS-UPDRS): Scale presentation and clinimetric testing results. Mov Disord 23:2129–2170. https://doi.org/10.1002/mds.22340

3.	Nasreddine ZS, Phillips NA, Bédirian V, et al (2005) The Montreal Cognitive Assessment, MoCA: a brief screening tool for mild cognitive impairment. J Am Geriatr Soc 53:695–699. https://doi.org/10.1111/j.1532-5415.2005.53221.x

4.	Matteau E, Dupré N, Langlois M, et al (2011) Mattis Dementia Rating Scale 2: Screening for MCI and Dementia. Am J Alzheimers Dis Other Demen 26:389. https://doi.org/10.1177/1533317511412046

5.	Hamilton M (1960) A rating scale for depression. J Neurol Neurosurg Psychiatry 23:56–62. https://doi.org/10.1136/jnnp.23.1.56

6.	Vassar SD, Bordelon YM, Hays RD, et al (2012) Confirmatory Factor Analysis of the Motor Unified Parkinson’s Disease Rating Scale. Park Dis 2012:719167. https://doi.org/10.1155/2012/719167

7.	Fasano A, Herzog J, Seifert E, et al (2011) Modulation of gait coordination by subthalamic stimulation improves freezing of gait. Mov Disord 26:844–851. https://doi.org/10.1002/mds.23583
